# Fine-tune regulation of carboxypeptidase N1 controls vascular patterning during zebrafish development

**DOI:** 10.1038/s41598-017-01976-x

**Published:** 2017-05-12

**Authors:** Ting-Yun Wu, Yi-Shan Wang, Yi-Chun Song, Zih-Ying Chen, Yi-Ting Chen, Chien-Chih Chiu, Chang-Yi Wu

**Affiliations:** 1 Department of Biological Sciences, National Sun Yat-sen University, Kaohsiung, Taiwan; 2 Institute of Medical Science and Technology, National Sun Yat-sen University, Kaohsiung, Taiwan; 3 Doctoral Degree Program in Marine Biotechnology, National Sun Yat-sen University and Academia Sinica, Kaohsiung, Taiwan; 4 Department of Biotechnology, Kaohsiung Medical University, Kaohsiung, Taiwan

## Abstract

Vascular development is regulated by complicated signals and molecules in vertebrates. In this study, we characterized a novel function of carboxypeptidase N1 (Cpn1) in the vasculature. We show that *cpn1* mRNA is expressed in developing vessels. The knockdown of *cpn1* by morpholino injection impairs the growth of intersegmental vessels (ISV) and caudal vein plexus (CVP), suggesting the role of *cpn1* in vascular development. We showed that vascular defects are not caused by cell death but are due to the impairment of migration and proliferation. Consistent with vascular growth defects, loss of *cpn1* affects the expression of the vascular markers *flt4*, *mrc1*, *flk*, *stabilin*, and *ephrinb2*. Furthermore, the overexpression of *cpn1* impaired the growth of ISV and CVP, but the remodeling expression of vascular markers was different from the knockdown of *cpn1*, indicating the differential regulation mechanisms in *cpn1*-overexpressing embryos. We examine the interaction between *cpn1* and multiple signals and observed that *cpn1* is regulated by Notch/VEGF signals for ISV growth and likely regulates BMP signals for CVP patterning. In conclusion, we demonstrate that *cpn1* has a critical role in the vascular development of zebrafish. We also reveal a fine-tune regulation of *cpn1* that controls vascular patterning mediated by multiple signals.

## Introduction

The development of vertebrate embryos depends on a precisely patterned, integrated network of blood vessels that supply oxygen and nutrients. The vascular system is one of the first organs that functions during vertebrate development. In addition, the formation of a precisely patterned, coordinated network of blood vessels is essential for embryonic survival. Blood vessels grow through vasculogenesis (*de novo* development of the vasculature) and angiogenesis (growth of blood vessels from existing vessels through sprouting and elongation)^1, 2^. Angiogenesis is the predominant mode of vessel growth and morphogenesis after the formation of the primary capillary plexus^3^. Although most vascular endothelial cells in adults are non-mitotic, they can be induced to enter the cell cycle and grow in specific physiological and pathological conditions.

The zebrafish model has been successfully used to elucidate molecular mechanisms underlying blood vessel formation during embryogenesis, including initial angioblast specification, artery and veindifferentiation, vessel formation, and localized patterning and morphogenesis of the vasculature^4–9^. Arteries and veins are formed from angioblast progenitors and are genetically pre-specified. After the formation of arteries and veins, angioblasts undergo further proliferation and migration to form a patterned network of small vessels, such as the growth of the trunk intersegmental vessels (ISVs) and caudal vein plexus (CVP) in zebrafish, which makes it ideal for investigating angiogenesis^8^. As the vessels migrate, leading tip cells take an active role in sensing the environment for guidance cues, whereas nonmigratory stalk cells lumenize and lose filopodia^10–12^. In addition to their embryonic role in angiogenesis, tip cells are involved in new vessel growth in many diseases. Thus, genes modulating the behavior of tip cells might have clinical importance as pharmaceutical targets.

Despite growing knowledge of the pathways leading to artery specification and ISV growth, little is known about the molecular mechanisms leading to vein specification, tip–stalk cell specification, and CVP patterning. Current evidence suggests that VEGFC/VEGFR3 (flt4) signaling is essential for vein identity and ISV tip cell identity mediated by VEGFA/VEGFR2 signal and inactivation of Notch signal^13, 14^, and BMP signaling is involved in CVP formation^15–17^. Our recent studies found that transcription factors Isl2 and Nr2f1b are crucial novel determinants of vein identity, ISV growth, and CVP patterning^18, 19^. Our Isl2/Nr2f1b transcriptomal analysis showed *cpn1*, that encodes the carboxypeptidase N subunit 1, is highly responsive and we hypothesized that *cpn1* plays a potential role in the vascular development. Carboxypeptidase N is a plasma metalloprotease and belongs to the peptidase M14 family. Carboxypeptidase N is composed of 2 catalytic activity (cpn1) and 2 regulatory (cpn2) subunits, and is essential for zebrafish liver development^20^. In addition, Davis *et al.* reported that carboxypeptidase N acts as an enzyme responsible for the C-terminal cleavage of stromal cell-derived factor-1 alpha (SDF-1a) in the circulation^21^. SDF-1a and its receptor CXCR4 have been shown to be critical for hematopoiesis, cardiovascular function, and angiogenesis^22^. However, the role of Cpn1 in vascular function has yet to be reported.

In this study, through *in-situ* hybridization, we showed that *cpn1* mRNA is expressed in developing vessels. The knockdown of *cpn1* by morpholino injection impaired the growth of ISV and CVP. In addition, *cpn1* overexpression caused vascular defects, suggesting the role of *cpn1* in controlling the growth of ISV and CVP. Thus, we propose a fine-tuned regulation of *cpn1* that controls vascular patterning. Furthermore, we explore the regulation of *cpn1* mediated by VEGF and Notch signals to control ISV growth and observe that *cpn1* contributes to CVP formation through interaction with BMP signals.

## Results

### *Cpn1* is conserved and *cpn1* mRNA is expressed in developing vessels

In our previous study, we identified the function of the transcription factors Isl2 and Nr2f1b in the vasculature mediated by Notch signals^18, 19^. Our Isl2/Nr2f1b transcriptomal analysis suggested that *cpn1* acts a potential function in the vasculature. In this study, we characterized a novel function of *cpn1* that contributes to vessel growth and patterning. *Cpn1* encodes carboxypeptidase, a plasma metalloprotease; however, no study has yet reported the role of *cpn1* in vascular development. Thus, we aligned the sequences of zebrafish Cpn1 with those of the humans (*Homo sapiens*), mice (*Mus musculus*), and chickens (*Gallus gallus*) using ClustalW2 software. The sequence alignment revealed that the zebrafish Cpn1 protein contains 450 amino acids with the N-terminal M14 family of the metallocarboxypeptidase domain and C-terminal peptidase-associated domain (sFig. 1A,B). A phylogenetic analysis of the amino acid sequences of Cpn1 proteins from different species demonstrated that the similarity of the amino acid sequences of zebrafish Cpn1 to those of humans, mice, and chickens are 69.3, 65.8, and 67.3, respectively; which is highly conserved among these vertebrates (sFig. 1C).

**Figure 1:**
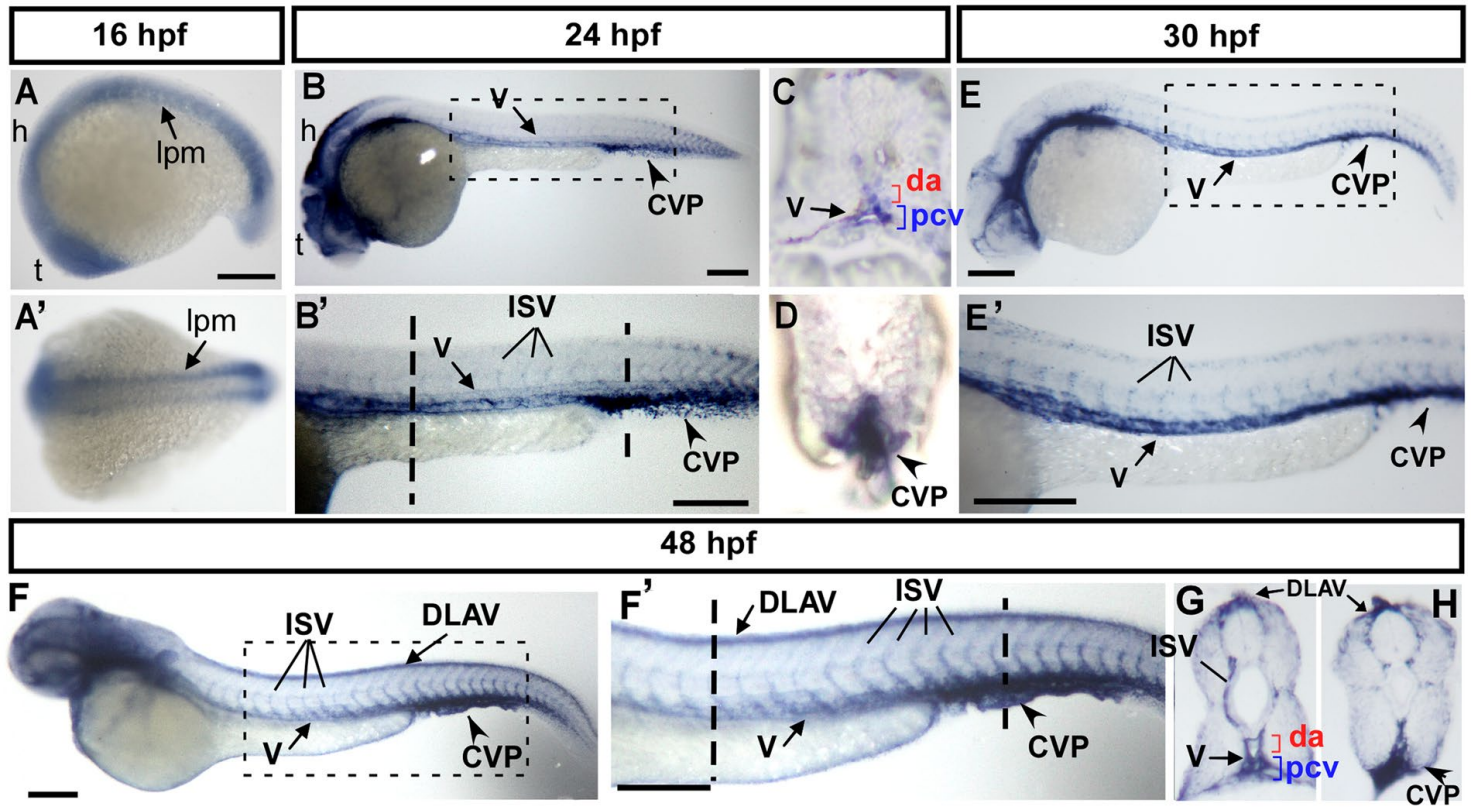
Spatiotemporal expression of *cpn1* during zebrafish development. (**A**) The lateral view shows *cpn1* mRNA expression at the 18 S stage in the lateral plate mesoderm (l pm), hindbrain (h), and telencephalon (t). (A′) The dorsal view of the embryos shows *cpn1* expression in the lpm. (**B**,B′) At 24 hpf, *cpn1* was expressed in the vessels (v), intersegmental vessels (ISV) and caudal vein plexus (CVP) of the trunk. B′ is an enlargement of B. (**C**,**D**) The cross sections of embryos from B′ demonstrate that *cpn1* is expressed in the dorsal aorta (da), posterior cardinal vein (pcv) and CVP. (**E**,E′) At 30 hpf, *cpn1* was expressed in the vessels (v), ISV, and CVP of the trunk. E′ is an enlargement of E. (**F**,F′,**G**,**H**) At 48 hpf, *cpn1* was expressed in ISVs, dorsal longitudinal anastomotic vessels (DLAV), vessels (v), da, pcv and CVP, as observed in the lateral view and transverse sections of the embryo trunk and tail regions. F′ is an enlargement of F. Scale bars in all figures represent 200 µm.

To explore the potential role of *cpn1* in the vascular development of zebrafish, we examined the expression pattern of *cpn1* mRNA during zebrafish development through *in situ* hybridization. We observed *cpn1* expression in 15–18 S stages in the lateral plate mesoderm (lpm), hindbrain (h), and telencephalon (t) (Fig. 1A,A′). At 24 hpf, *cpn1* was expressed in the vessels (both dorsal aorta (da) and posterior cardinal vein (pcv)), ISV and CVP, as depicted in the lateral view or cross-section (Fig. 1B–D,B′). *Cpn1* expression was continuously observed in the vessels (v), ISV, CVP, and dorsal longitudinal anastomotic vessel (DLAV) of the trunk from 30–48 hpf, as shown in the lateral view and transverse sections of the embryo trunk and tail regions (Fig. 1E–G,E′,F′). The spatiotemporal expression of *cpn1* in developing vessels suggests the role of *cpn1* in the vascular development.

### Knockdown of *cpn1* causes vascular defects during zebrafish development

**Figure 2:**
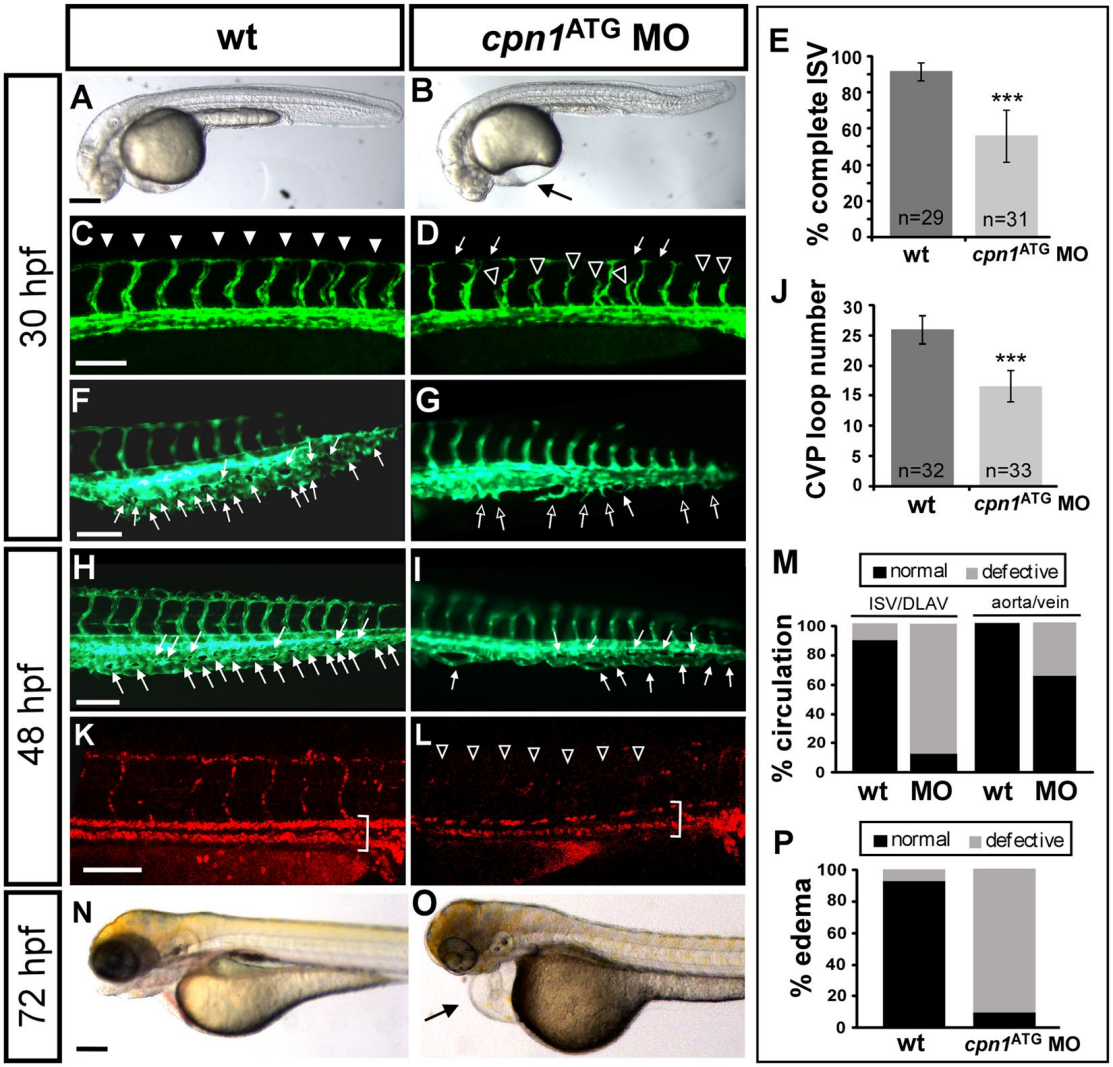
Knockdown of *cpn1* causes vascular defects in zebrafish development. (**A**,**B**) The bright field images of control and *cpn1* ^ATG^ morpholino (3.41 ng)-injected embryos at 30 hpf. (**C**–**J**) At 30 hpf, ISVs reached DLAV and formed a honeycomb-like structure in the caudal vein plexus (CVP) in wild-type controls by using *Tg* (*fli1:eGFP* ^*y1*^) zebrafish (**C**,**F**). Compared with wild-type control (arrowheads in C, arrows in F), loss of *cpn1* caused ISV growth defects (hollow arrowheads in D) and less honeycomb-like structure formation in CVP (arrows in G). At 48 hpf, less CVP loop formation (arrows in I) compared with that in control (arrows in H). (**K**,**L**) The injection of *cpn1* ^ATG^ MO into *Tg* (*fli1a:eGFP* ^*y1*^; *gata1*:*dsRed* ^*sd2*^) embryos revealed that loss of *cpn1* caused circulation defects in ISVs at 48 hpf. (**N**,**O**) At 72 hpf, *cpn1* ^ATG^ morphants had pericardial edema (arrow in O). (**E**) The percentage of completed ISVs decreased by approximately 45% in *cpn1* ^ATG^ morphants (n = 29 in wt and n = 31 in *cpn1* ^ATG^ MO) at 30 hpf. (**J**) The loop formation in CVP exhibited a decrease in *cpn1* ^ATG^ morphants (n = 32 in wt and n = 33 in *cpn1* ^ATG^ MO) at 48 hpf. (**M**) Quantification data showed *cpn1* morphants had approximately 90% defects in the ISV–DLAV circulation and 40% defects in the aorta–vein circulation compared to controls (n = 39 in control and n = 28 in *cpn1* MO) at 48 hpf. (**P**) The percentage of pericardial edema was 90% in *cpn1* ^ATG^ morphants compared with that in controls (n = 127 in controls and n = 50 in *cpn1* ^ATG^ MO) at 72 hpf. These results were confirmed by three independent experiments. Data are represented as means ± S.D. ***Refers to p < 0.0001 by an unpaired Student’s *t*-test. The scale bar is 200 µm for A, B, N, and O, and 100 µm for C–L.

To identify the role of *cpn1* in vascular development, we used *Tg* (*kdrl*:*eGFP*)^*la116*^ embryos expressing GFP in endothelial cells. After the knockdown of *cpn1* through the administration of a morpholino injection into the embryos, we observed vascular defects in *cpn1* morphants. We knocked down *cpn1* expression by injecting 3.4 ng of ATG morpholino to block the translational site (*cpn1* ^ATG^ MO). The loss of *cpn1* caused the following 2 marked vascular phenotypes: ISV growth defects and CVP mispatterning. Compared with 90% complete ISVs in uninjected controls (n = 29 embryos), we observed only 45% complete ISVs (n = 31 embryos) with slightly edema in *cpn1* ^ATG^ morphants at 30 hpf (Fig. 2A–E). In addition, we observed defects in CVP formation as a second phenotype. From 30–48 hpf in wt fish, endothelial cells in the CVP region underwent angiogenic sprouting and migration, and formed a honeycomb structure or a loop (Fig. 2F–I). The sprouting and loop structure formation in CVP was decreased in the *cpn1* morphants as compared to the uninjected controls at 30 hpf (Fig. 2F,G); and the honeycomb-like loop at CVP was quantified at 48 hpf (n = 32 in wt and n = 33 in *cpn1* morphants; Fig. 2H–J). These data indicate that *cpn1* may play a critical role in controlling the behavior of endothelial tip cells and in the regulation of ISV and CVP formation during angiogenesis. Furthermore, since pericardial edema and circulation blockage are common secondary effects of blood vessel impairment, we examined whether these side effects occurred in *cpn1* morphants. We observed limited blood flow in the trunk of *cpn1* morphants at 48 hpf (Fig. 2K,L). Approximately 60% *cpn1* morphant embryos had circulation in the axial vessels and only about 10% *cpn1* morphant embryos had circulation in ISVs (Fig. 2M, n = 39 in control and n = 28 in *cpn1* ^ATG^ MO). At 72 hpf, *cpn1* morphants showed an increasing edema ~90% compared to the controls (Fig. 2N–P, n = 127 in control and n = 50 in *cpn1* ^ATG^ MO), which was likely caused by circulation defects. The data are consistent with vascular defects in the knock-downed *cpn1* embryos.

### Specificity of *cpn1* knockdown by morpholino

We observed that the loss of *cpn1* causes vascular defects by targeting the translation initiation site (*cpn1* ^ATG^ MO; Fig. 2). To investigate whether the phenotype of *cpn1* morpholino knockdown is loss-of-cpn1 specific, we examined the effects of additional morpholino blocking the splicing site at the exon1–intron1 boundary (*cpn1* ^e1i1^ MO). Our results showed similar vascular defects in *cpn1* ^e1i1^ MO (sFig. 2) compared to *cpn1* ^ATG^ morphants. We observed and quantified vascular defects in CVP formation and ISV growth in *cpn1* ^e1i1^ MO at 30hpf and 48hpf (sFig. 2C–J). Furthermore, we examined the efficiency of *cpn1* ^e1i1^ morpholino knockdown. The RT-PCR analysis revealed that the injection of 5.1 ng or 15.8 ng of *cpn1* ^e1i1^ morpholino dose-dependently reduced the normal fragment of *cpn1* (sFig. 2K,L), indicating that the loss of *cpn1* expression is caused by morpholino inhibition. The data suggests that the phenotype of *cpn1* morpholino knockdown is specific. Furthermore, to test sequence targeting specificity of *cpn1* ^ATG^ morpholinos, we made a *cpn-atg-GFP* fusion construct containing the *cpn1* ^ATG^ MO targeting sequence. Embryos co-injected with mRNA from the *cpn1-atg-GFP* construct and *cpn1* ^ATG^ MO reduced and/or blocked the GFP signal in the embryos at 24 hpf (sFig. 3A,B), which suggests the specificity of ATG morpholino targeting. To confirm the efficacy of morpholino knockdown, we performed a Western blot analysis and showed a significant reduction of Cpn1 protein levels in both ATG and splicing morpholinos injected embryos (sFig. 3C). These data are consistent with the efficacy and specificity of *cpn1* knockdown by morpholino.

Moreover, injection of 10 ng control morpholino did not cause vascular defects in ISV and CVP (sFig. 4A,B), which is consistent with the phenotypic specificity of morpholino knockdown of *cpn1*. We also assessed the gross developmental process in *cpn1* morphants at 24–25 hpf and showed development in other tissue/organs is unaffected because the knockdown of *cpn1* did not alter the expression of *cmlc2* (heart marker), *gata6* (heart and gut marker), *myoD* (somite marker), and *sox3* (neural markers) (sFig. 5A–H). Meanwhile, we measured the heart rate by direct visual examination of ventricle beating in embryos at 24–25 hpf and found no difference between the uninjected controls and *cpn1* morphants (sFig. 5I), suggesting the knockdown of *cpn1* did not cause developmental delay.

### Knockdown of *cpn1* impairs the growth of ISV cells

**Figure 3:**
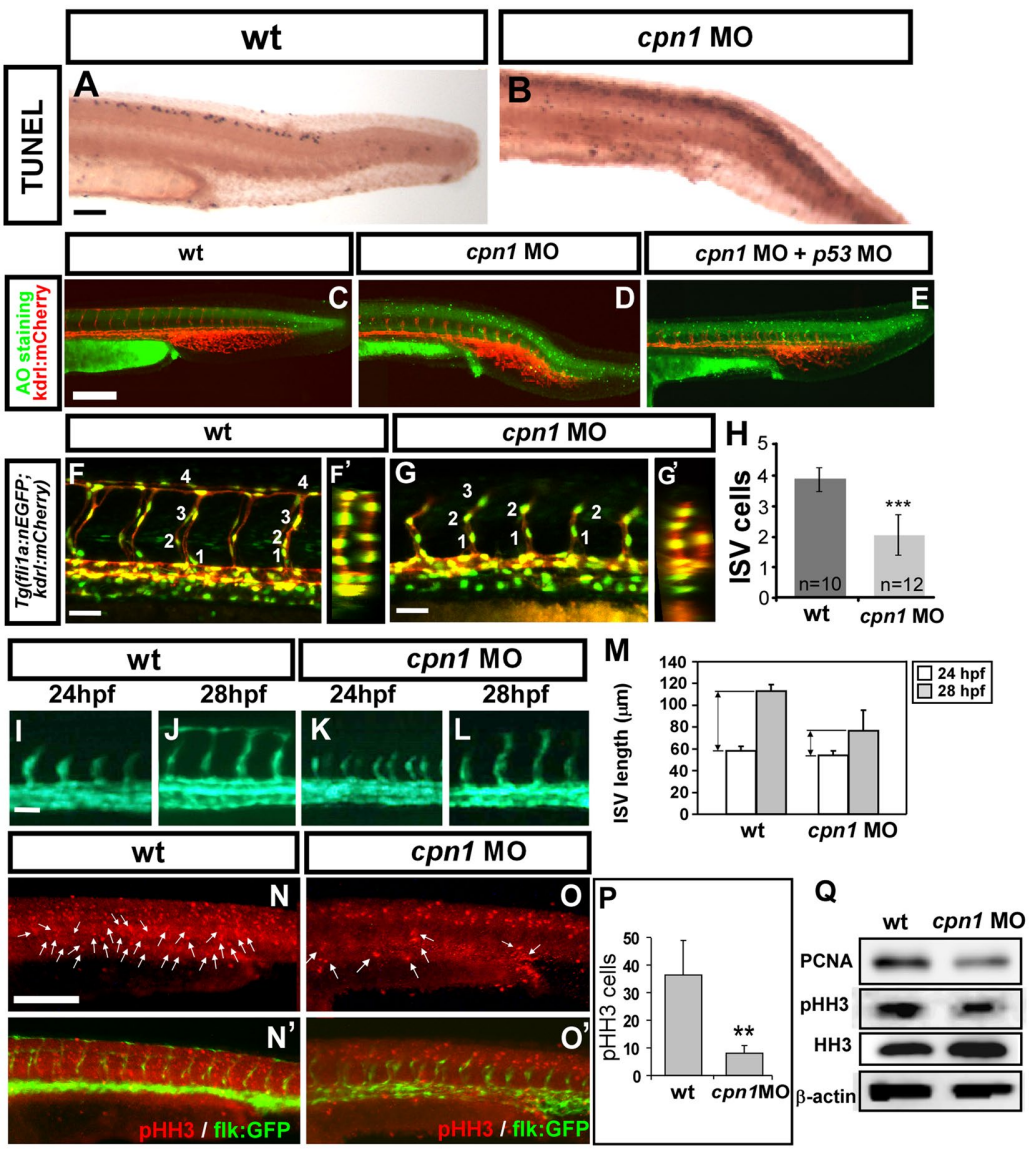
Knockdown of *cpn1* impairs the growth of ISV cells. (**A**–**E**) TUNEL assay and acridine orange (AO) staining was used to detect apoptotic cells in wt and *cpn1* morphants. (**A**,**B**) Apoptotic cells (black dots) were observed in the dorsal region, but not in the vasculature in *cpn1* morphants. (**C**–**E**) Representative images of AO staining in uninjected control (**C**), *cpn1* morphants (**D**) and *cpn1* MO co-injected with *p53* MO (**E**) on *Tg*(*kdrl:mCherry*)^*ci5*^ fish. Some increased apoptotic cells were observed on the skin and at the epidermis of the tail region in *cpn1* MO (**D**), but not in the vasculature as compared to controls (**C**). Co-injection of *cpn1* MO with *p53* MO showed a reduction of apoptotic cells but still caused vascular defects in ISV and CVP (E). (**F**,F′,**G**,G′) Lateral and cross-sectional views of confocal images of *Tg*(*kdrl*:*mCherry* ^*ci5*^; *fli1a*:*nEGFP* ^*y7*^) embryos with *cpn1* MO injection (**G**,G′) and uninjected control (**F**,F′) at 30hpf. (**H**) Quantification of the average number of cells per ISV in wt (n = 10) and *cpn1* MO (n = 12) at 30 hpf. (**I**–**L**) Migration assay is used to measure the difference of ISV length from 24 hpf to 28 hpf in wt control and *cpn1* MO (n = 6 in wt and morphants), and quantitative results are shown in M. (**N**,N′,**O**,O′) Proliferation marker pHH3 from immunofluorescent image was counted in the trunk region beneath the neural tube and above yolk extension area, which is related to the regions of the main vessels and ISVs. (**P**) The number of mitotic cells (pHH3 cells) in control was 36.4 ± 12.4 (n = 8) and in *cpn1* MO was 8 ± 2.9 (n = 6). (**Q**) Western blot analysis showed a reduced expression level of phosphor-histone H3 (pHH3) and another proliferation marker PCNA. β-actin and histone H3 serve as loading controls. Scale bars are 200 μm for (**A**–**E**,**N**,**O**, N′,O′) and 50 μm for (**F**,**G**,**I**–**L**). All results were performed by two independent experiments and data are represented as means ± S.D. ***Refers to *p* < 0.0001 and **refers to *p* < 0.001 by an unpaired Student’s *t*-test.

The knockdown of *cpn1* caused vascular growth defects, suggesting the interruption of endothelial cell behaviors such as migration, proliferation, or an increase of cell death. To test these possibilities, we first performed a TUNEL assay and Acridine Orange (AO) staining to detect apoptotic cells. We observed *cpn1* morphants have an increase in apoptotic cells at the epidermis of dorsal region compared to wild-type embryos; however, morpholino-induced cell death did not show in the vessel region of the trunk (Fig. 3A–D). In addition, co-injection of *cpn1* MO with *p53* morpholino showed a reduction of apoptotic cells but still caused vascular defects in ISV and CVP (Fig. 3E). The data suggest that vascular defects are not due to endothelial cell death.

We investigated whether the loss of *cpn1* affects cell proliferation by determining the number of endothelial cells per ISV in *Tg* (*kdrl*:*mCherry* ^*ci5*^; *fli1a*:*negfp* ^*y7*^) embryos. The loss of *cpn1* showed reduced ISV cells significantly compared to wild-type controls (Fig. 3F–G,F′,G′, H n = 12 in *cpn1* morphants and n = 10 in wt control, *p* < 0.0001). In addition, only a small number of ISV cells could reach the top of the embryo to form DLAV, suggesting the impairment of endothelial cell migration in *cpn1* morphants.

To confirm a migration defect, we measured the difference of ISV length from 24–28 hpf in the control and *cpn1* MO. The difference of ISV length in *cpn1* MO is significantly shorter than in the control, indicating the migration ability is impaired (Fig. 3I–M). We further demonstrated the proliferation defect in *cpn1* MO by examining the expression levels of proliferation marker phosphorylated form of histone H3 (pHH3) and proliferating cell nuclear antigen (PCNA). Immunostaining with proliferation marker anti-phosphohistone 3 antibody recognizes mitotic cells and the amount of mitotic cells in the trunk of *cpn1* MO showed fewer red spots compared to the control (arrows in Fig. 3N,O) and quantitative results in Fig. 3P. In addition, the Western blotting analysis showed pHH3 and PCNA signals are reduced in *cpn1* MO (Fig. 3Q), suggesting a reduction of cell proliferation. Together, the data suggest that *cpn1* is essential for endothelial cell growth for patterning ISV and CVP, likely through the regulation of proliferation and migration of the cells.

### Knockdown of *cpn1* modulates the expression of vascular markers

**Figure 4:**
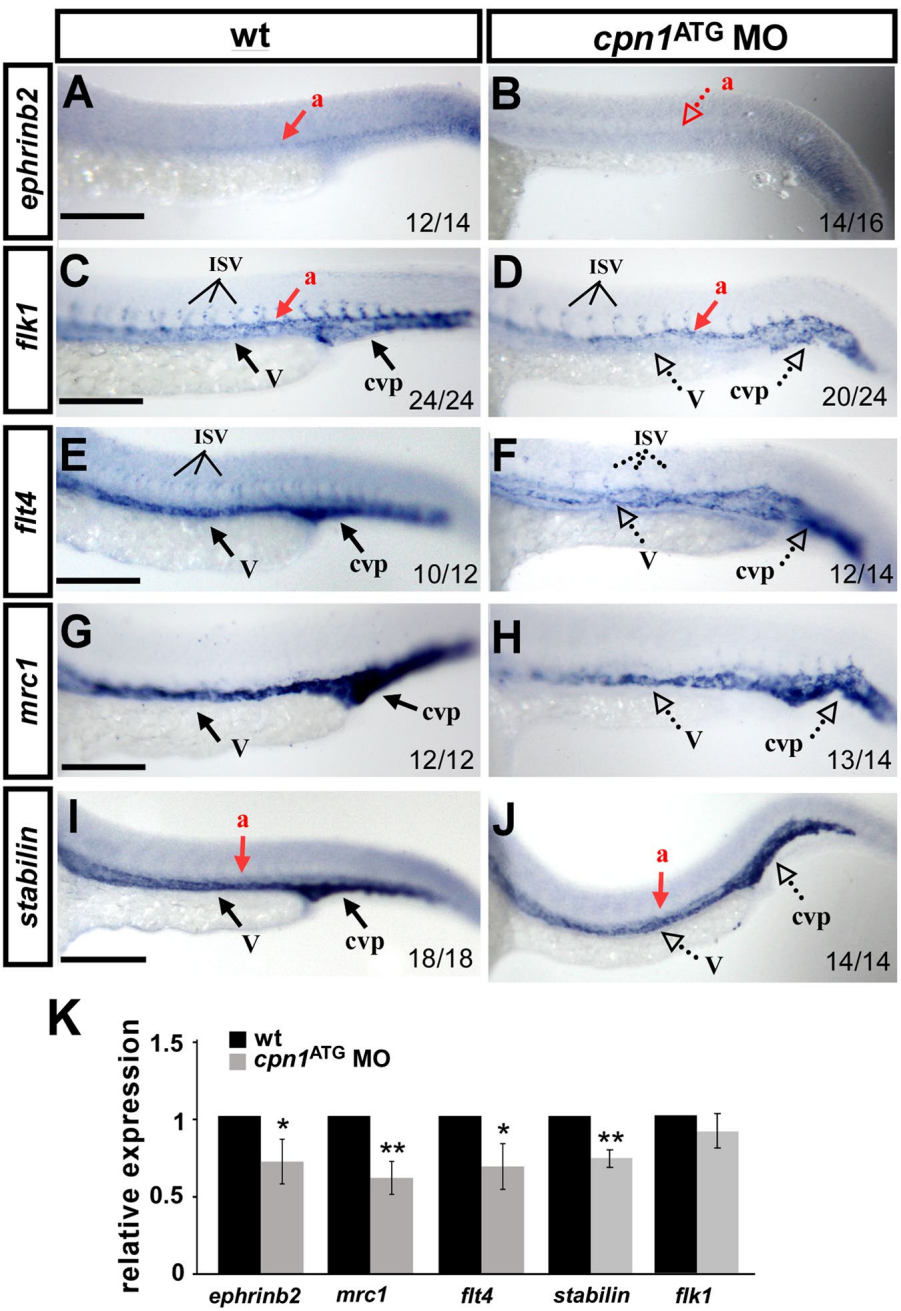
Knockdown of *cpn1* modulates the expression of vascular markers. Compared with wild-type (wt) controls (**A**,**C**,**E**,**G**,**I**), *cpn1* ^ATG^ morphants had decreased expression of the arterial marker *ephrinb2* (**B**), venous markers *mrc1* (**H**) and *flt4* (**F**), and pan-vascular markers *flk* (**D**) and *stabilin* (J). dorsal aorta (a); vein (v); intersegmental vessels (ISV) and caudal vein plexus (cvp). The values on the bottom indicate the number of embryos exhibiting phenotype per total number of embryos analyzed from two independent experiments. (**K**) qPCR assay showed the relative expression level of *ephrinb2* (0.71 ± 0.12), *mrc1*(0.61 ± 0.11), *flt4* (0.69 ± 0.15), *satbilin* (0.74 ± 0.06) and *flk1*(0.9 ± 0.11) in *cpn1* ^ATG^ morphants and in wt controls. qPCR data are represented as means ± S.D. **Refers to p < 0.01 and *refers to p < 0.05 according to the unpaired Student’s *t*-test. Scale bars are 200 µm.

The growth defects in ISV and mispatterning in CVP after the loss of *cpn1* suggest that *cpn1* is essential for vascular development and likely plays a role in modulating vascular identity. To test this hypothesis, we examined the expression of the vascular markers *flk1*, *flt4*, *mrc1*, *stabilin*, and *ephrinb2* through *in situ* hybridization and qPCR analysis. We observed that the expression of the venous/ISV markers *flt4* and *mrc1* was decreased in *cpn1* morphants compared to wt controls at 24 hpf (Fig. 4). The expression of the arterial marker *ephrinb2* and the pan-vascular markers *flk1* and *stabilin* were also reduced by *in-situ* hybridization. To determine the extent of the decrease in the marker expression, we quantified *flt4*, *ephrinb2*, *mrc1*, stabilin, and *flk1* transcript levels through qPCR and determined a 30–40% decrease in the expression in *cpn1* morphants, except *flk1* (slightly but not significantly decreased). These results suggest that *cpn1* regulates several vascular genes to promote vessel formation.

### Overexpression of *cpn1* also causes vascular defects in zebrafish embryos

**Figure 5:**
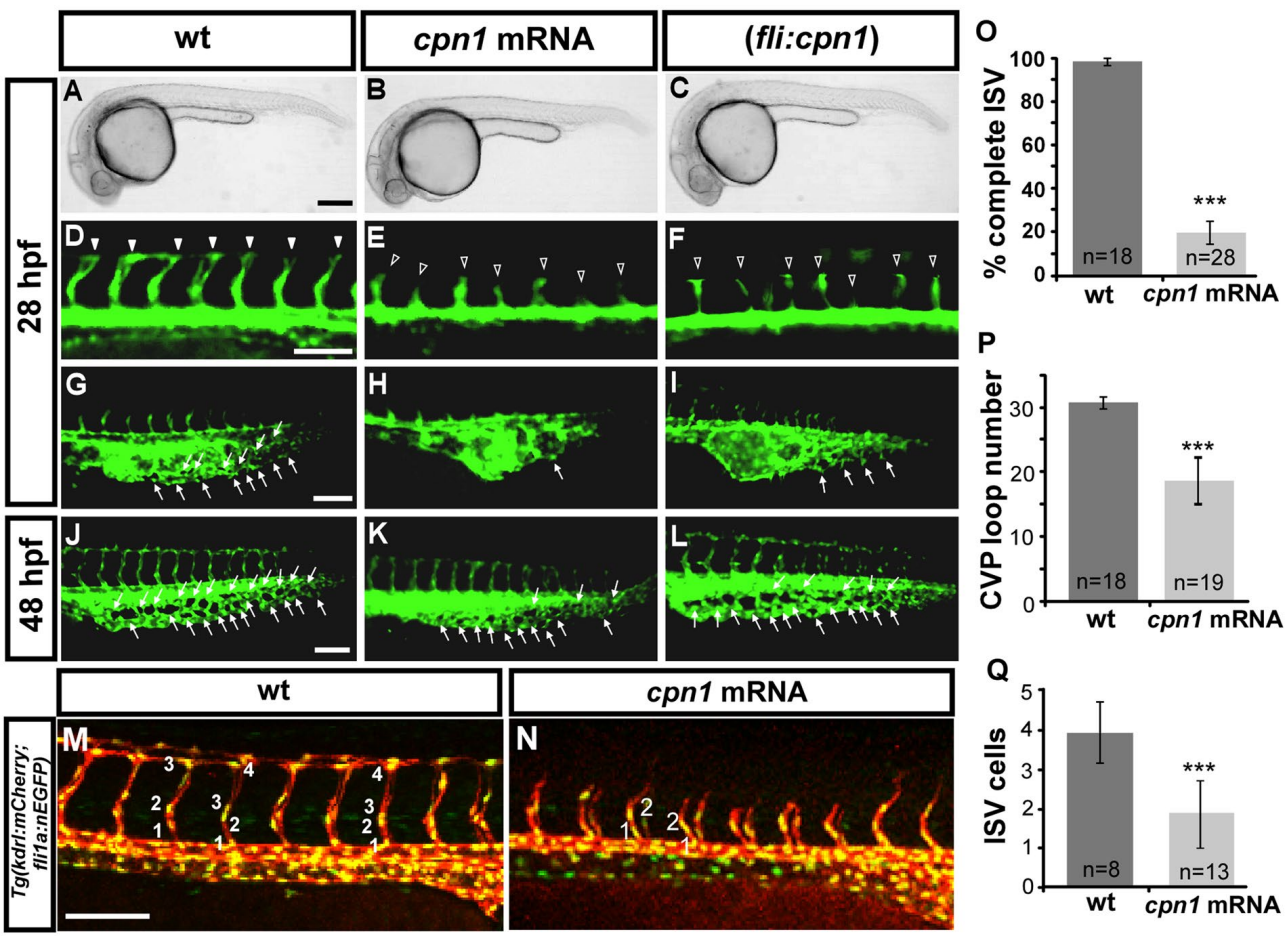
Overexpression of *cpn1* causes vascular defects in zebrafish embryos. (**A**–**C**) The bright field images of uninjected control and *cpn1* mRNA-injected (0.93ng) and (*fli1*:*cpn1*) embryos at 28 hpf. Compared with controls (arrowheads in (**D**), arrows in (**G**), the overexpression of *cpn1* caused ISV growth defects (hollow arrowheads in (**E**, **F**) and less honeycomb-like structure formation in CVP at 28 hpf (**H**,**I**). At 28 hpf, ISVs reached DLAV and formed a honeycomb-like structure in CVP in controls. At 48 hpf, CVP loop formation was less (arrows in K, L) than in controls (arrows in J). (**M**,**N**) Injection of *cpn1* mRNA into *Tg(kdrl:mCherry* ^*ci5*^; *fli1a:nEGFP* ^*y7*^) embryos (**N**) shows that the cell number per ISV was lower than in control (**M**) and ISVs did not reach DLAV as compared to control at 28hpf. (**O**) The percentage of completed ISVs decreased by approximately 80% in *cpn1* mRNA injected embryos (n = 18 in wt and n = 28 in *cpn1* mRNA) at 28 hpf. (**P**) The loop formation at CVP decreased in *cpn1* mRNA injected samples (n = 18 in wt and n = 19 in *cpn1* mRNA) at 48 hpf. (**Q**) The average number of cells per ISV decreased in *cpn1* mRNA injected embryos (n = 8 in wt and n = 15 in *cpn1* mRNA) at 30 hpf. The images are the representative pictures from two independent experiments. Quantitative data are represented as means ± S.D. ***Refers to p < 0.0001 by an unpaired Student’s *t*-test. Scale bars are 200 µm for A–C and 100 µm for D–N.

Because the loss of *cpn1* caused vascular defects, we hypothesized that *cpn1* overexpression might promote vascular growth. We overexpressed *cpn1* by injecting 0.3~0.93 ng of mRNA (represent as *cpn1* mRNA) into transgenic *Tg* (*kdrl*:*EGFP*) embryos or overexpressed *cpn1* under the control of the *fli1a* promoter in transgenic embryos (represent as (*fli*:*cpn1*)). Overexpression of *cpn1* showed normal morphology at 28 hpf (Fig. 5A–C). Surprisingly, the overexpression of *cpn1* resulted in ISV growth defects and CVP mispatterning (Fig. 5D–L). While injection of *cpn1* mRNA into embryos, a high percentage of ISV showed stalling growth at the mid-somite and did not reach DLAV compared to uninjected controls at 28 hpf (Fig. 5D,E). Quantitative results showed over 95% complete ISVs in uninjected controls (n = 18 embryos) and only ~20% complete ISVs (n = 28 embryos) in *cpn1* mRNA-injected embryos (Fig. 5O). In addition, we observed defects in CVP formation in *cpn1*-overexpressing embryos. Compared with uninjected controls in CVP region from 28–48 hpf, we observed less or no endothelial cell sprouting at 28 hpf and less honeycomb-like capillary loop formation at 48 hpf (Fig. 5G–L). The quantification of CVP loop formation showed a decrease in *cpn1* mRNA embryos (n = 19) at 48 hpf (Fig. 5P). In addition, injection of *cpn1* mRNA into *Tg(kdrl:mCherry* ^*ci5*^; *fli1a:nEGFP* ^*y7*^) embryos shows that the cell numbers per ISV was lower and the ISVs did not reach DLAV when compared to uninjected wt control at 30 hpf (Fig. 5M,N,Q). We evaluated whether *cpn1* overexpression in embryos increases cell death. The TUNEL assays revealed no significant apoptotic cells in *cpn1*-overexpressing fish compared with that in wt controls (data not shown). Together, the data suggest that *cpn1* overexpression can disrupt endothelial cell growth for vascular patterning, likely through the regulation of cell proliferation and migration.

Consistent with the defects of the vasculature in *cpn1*-overexpressing embryos, we observed that *cpn1* overexpression also causes circulation defects and pericardial edema (sFig. 6). The injection of *cpn1* mRNA into *Tg* (*fli1a*:*eGFP* ^*y1*^; *gata1*:*dsRed* ^*sd2*^) embryos resulted in circulation defects observed as limited blood flow in the aorta-vein and ISV-DLAV at 48 hpf (sFig. 6A–C). Furthermore, compared with controls (n = 33), 70% *cpn1* mRNA-injected embryos (n = 28) had pericardial edema at 72 hpf (sFig. 6D–F). Because both loss or gain of *cpn1* function causes vascular defects, *cpn1* may have a critical role in controlling ISV and CVP formation and thus should be fine-tune regulated.

Although the overexpression of *cpn1* impairs vascular development, we found embryos with a low level of *cpn1* mRNA (<200 pg) injection did not show vascular defects. Thus, we can perform a rescue experiment to confirm whether the vascular defects were specifically caused by morpholino knockdown. We injected 100 pg *cpn1* mRNA in the control embryos and *cpn1* ^e1i1^ morphant and found that the overexpression of *cpn1* mRNA in *cpn1* ^e1i1^ MO restores ISV growth by 35% compared to the injection of *cpn1* morpholino alone (*cpn1* ^e1i1^ MO) at 30 hpf (sFig. 7A–E), while the overexpression of *cpn1* in control embryos had no obvious effect on the vascular development (sFig. 7B) compared to the uninjected controls (sFig. 7A). The data specifically confirm that the knockdown of *cpn1* impaired vascular development.

### Gain of *cpn1* function alters the expression of vascular markers

The overexpression of *cpn1* causes ISV growth defects and CVP mispatterning, suggesting that the overexpression of *cpn1* can modulate vascular identity. To test this hypothesis, we examined the expression of the vascular markers *flk1*, *flt4*, *mrc1*, *stabilin*, and *ephrinb2* through the *in situ* hybridization or qPCR. We observed that the expression of the venous/ISV markers *flt4, mrc1*, and *dab2* was lower in *cpn1*-overexpressing fish than in wt controls at 24 hpf; whereas the expression of the arterial marker *ephrinb2* remained unchanged. (sFig. 8A–F,K). In addition, the expression of the pan-vascular markers *flk* and *stabilin* at the vessels and CVP was no-to-slightly increased in embryos (sFig. 8G–J). These results suggest that overexpression of *cpn1* has a higher impact on venous genes than on arterial genes to regulate vascular development. The remodeling expression pattern of vascular markers was different from that after *cpn1* knockdown, indicating that differential mechanisms that control vascular development in *cpn1*-overexpressing endothelial cells.

### Carboxypeptidase activity is crucial for vascular development

**Figure 6:**
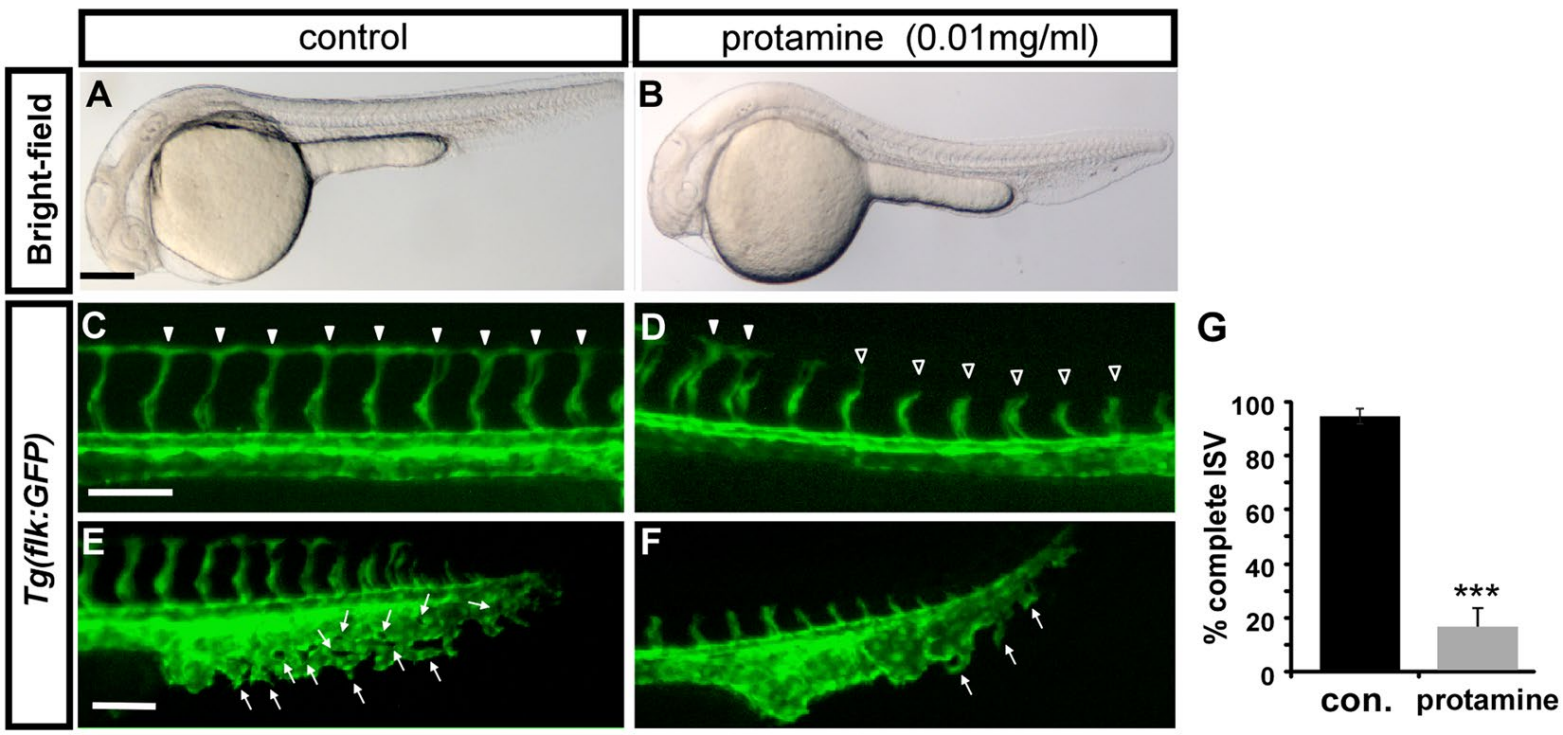
Inhibition of carboxypeptidase activity causes vascular defects. (**A**,**B**) Bright-field images of untreated control and embryos treated with 0.01 mg/ml protamine, a carboxypeptidase inhibitor. (**C**–**F**) The protamine-treated embryos showed stalling of ISVs (**D**) and mispatterned CVP (**F**) compared with completed growth of ISV (arrowheads in C) and the sprouting or loop structure of CVP (**E**) in wild-type embryos at 30 hpf. (**E**) The quantification of complete ISVs in embryos treated with 0.01 mg/ml protamine (18 ± 7%, n = 10) and untreated controls (92 ± 3%, n = 10). The data shown is representative of two independent experiments. Data are represented as means ± S.D. ***Refers to *p* < 0.0001 by an unpaired Student’s *t*-test. Scale bars are 200 µm for A-B and 100 µm for C-F.

*Cpn1* is required for vascular development and *cpn1* encodes carboxypeptidase N peptide 1, the enzyme activity site, suggesting that carboxypeptidase activity is crucial for vascular development. The tetramers of carboxypeptidase N are composed of 2 Cpn1 active subunits and 2 Cpn2 regulatory subunits. To evaluate whether carboxypeptidase activity is crucial for vascular development, we inhibited carboxypeptidase N activity by using the protamine treatment^23^. After the addition of 0.01 mg/ml protamine into the fish medium, we found no morphological difference in the control and protamine-treated embryos (Fig. 6A,B); however, we observed stalling of ISVs with 74% decrease in ISV completion and CVP mispatterning in embryos (Fig. 6D,F) compared with that in untreated embryos (Fig. 6C,E). We confirmed that protamine treatment dose-dependently impairs vascular development (data not shown).

### Interaction among *cpn1* and multiple signals

**Figure 7:**
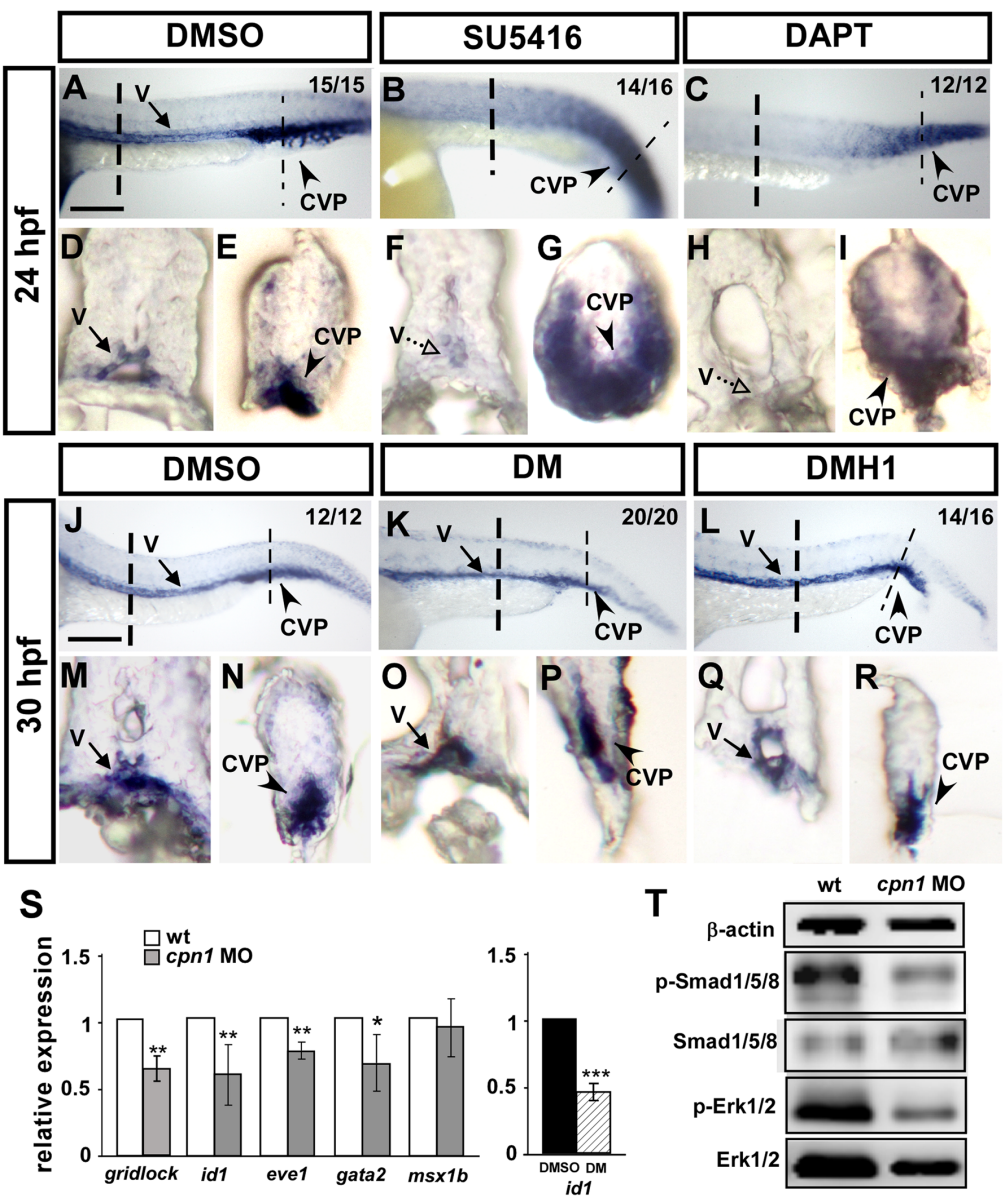
Interaction among cpn1, VEGF/Notch, and BMP signals. In the trunk region, the expression level of *cpn1* was decreased in SU5416- (**B**,**F**) and DAPT (**C**,**H**)-treated embryos. In the tail region, the ectopic expression of *cpn1* was observed in SU5416- (**B**,**G**) or DAPT-treated (**C**,**I**) embryos compared with that in DMSO-control embryos (**A**,**D**,**E**). (**D**–**I**) Transverse sections of the embryo trunk and tail region from A, B, and C. (**J**–**R**) Using DM and DMH1 to specify inactive BMP signals. In the trunk region, the expression level of *cpn1* remained unchanged after DM (**K**,**O**) or DMH1 (**L**,**R**) treatment compared with that in DMSO control (**J**,**M**). In the tail region, the expression of *cpn1* exhibited no differences in either DM (**K**,**P**) or DMH1 (**L**,**R**) treatment compared with that in DMSO controls (**J**,**N**). (**M**–**R**) Transverse sections of the embryo trunk and tail regions from J-L. Values on the top right indicate the number of embryos exhibiting phenotype per total number of embryos analyzed from 2 indepenedent experiments. Scale bars in A-C, J-L are 200 µm. (**S**) Relative expression of Notch downstream target *gridlock* and BMP regulated targets *id1, eve1, gata2* and *msx1b*. The expression of *id1* is downregulated while the BMP signal is blocked by DM treatment compared to DMSO as a positive control. Data are represented as means ± S.D. ***Refers to *p* < 0.001, **refers to *p* < 0.01 and *refers to *p* < 0.05 by an unpaired Student’s *t*-test. (**T**) Western blot analysis from two independent experiments showed reduced phosphorylation of Smad1/5/8 and ERK1/2 in *cpn1* morphants compared to control, and β-actin serves as a loading control.

We observed that the overexpression or knockdown of *cpn1* impaired ISV growth and caused CVP defects. Notch signaling is crucial for aorta-vein identity and ISV growth with the interaction of VEGFR2 signaling in the trunk of zebrafish. In addition, BMP signaling regulates angiogenic sprouting from the posterior cardinal vein to form CVP, which is a distinct mechanism of angiogenesis from ISV growth^15^. Thus, we investigated the regulatory relationship between *cpn1* and *Notch* or *VEGFR2*. We inactivated Notch and VEGFR2 signaling through exogenous DAPT and SU5416^24^ treatment, respectively (Fig. 7A–C). We observed that *cpn1* expression was downregulated when Notch or VEGFR2 signals were inhibited in the trunk area. Interestingly, in the tail region, the expression of *cpn1* was ectopic in SU5416 or DAPT-treated embryos. The transverse sections of the embryo trunk and the tail region confirmed this observation (Fig. 7D–I). The data suggest that *cpn1* controls vascular growth likely regulated by Notch and VEGF pathways in the trunk.

Wiley and co-workers reported that BMP signaling pathways regulate sprouting angiogenesis from the axial vein to form CVP, which provides a distinct mechanism of angiogenic sprouting. We examined whether *cpn1* interacts with BMP signal functioning in CVP formation by using DMH1 (4-(6-(4-isopropoxyphenyl) pyrazolo (1, 5-a) pyrimidin-3-yl) quinoline), which inhibits activin receptor-like kinase 2/3 and DM to inhibit BMP receptor 2 (BMPR2) signals^25^. Compared with the DMSO controls, the expression level of *cpn1* remained unchanged after DM or DMH1 treatment in the trunk and tail regions (Fig. 7J–L). The data suggest that *cpn1* is not regulated by BMP signals. Instead, we examined whether Cpn1 could regulate BMP signals. The qPCR analysis results showed that a decreased expression of the Notch downstream target *gridlock* and BMP regulated targets *id1, eve1* and *gata2*, suggesting the regulatory role of *cpn1* (Fig. 7S). In addition, the Western blot analysis showed the reduced phosphorylation of Smad1/5/8 (the main effectors of BMP signal), total and phosphorylated Erk1/2 in *cpn1* morphants compared to the controls (Fig. 7T). The decreased expression of *gridlock* mRNA and Erk1/2 protein suggest the Cpn1 controls ISV mediated with Notch/VEGF signals. The reduced mRNA expression of BMP targets and the phosphorylated level of Smad1/5/8 in *cpn1* MO indicate Cpn1 might regulate BMP signals to control CVP patterning.

In summary, the knockdown or overexpression of *cpn1* caused vascular defects, suggesting the role of *cpn1* in controlling ISV and CVP growth through a fine-tuned regulation. In addition, *cpn1* regulates vascular development mediated by distinct signals at different localizations in zebrafish.

## Discussion

This study shows that *cpn1* mRNA is expressed in developing vessels. The knockdown of *cpn1* through an injection of morpholino caused vascular defects, suggesting the role of *cpn1* in controlling ISV and CVP growth. We have demonstrated that vascular defects in *cpn1* morphants are caused by the impairment of migration and proliferation but not by nonspecific cell death. In addition, *cpn1* MO knockdown acted specifically and the expression level of vascular markers changed in *cpn1* MO. The results suggest that *cpn1* is essential for vascular development. Furthermore, the overexpression of *cpn1* impaired the growth of ISV and CVP by reducing ISV cell proliferation rather than cell death. The remodeling expression of vascular markers was different from the knockdown of *cpn1*, indicating that different mechanisms control vascular patterning in *cpn1*-overexpressing cells. Finally, we explored the regulation of *cpn1* mediated by VEGF and Notch signals in the trunk, which is crucial for ISV growth. In the tail region, *cpn1* functions in CVP formation likely through interacting with BMP signals. Together, we observed that loss and gain of *cpn1* function caused vascular defects and revealed a fine-tuned regulation of *cpn1* that controls vascular patterning mediated by multiple signals at different localizations in zebrafish.

Our previous studies found the vascular functions of Isl2 and Nr2f1b are mediated by Notch signaling^18, 19^. In this study, we demonstrated that *cpn1* functions in the vasculature are mediated by VEGF and Notch signals. Our transcriptomal analysis suggests that *cpn1* is likely regulated by Isl2/Nr2f1b. Interestingly, we determined several consensus binding sites of Isl2 or Nr2f located within the 5-kb upstream sequence of *cpn1* by using the sequence analysis software Regulatory Sequence Analysis Tool (RSAT). Future studies will examine the direct or indirect interaction of *isl2/nr2f1b* and *cpn1* genes. Because increased or decreased expression of *cpn1* can cause vascular defects, other regulators that control *cpn1* expression may be present in addition to Isl2 and Nr2f1b. Promoter sequence analysis can provide information on the crucial regulatory region or sequence and help in determining new regulators for the specific *cpn1* gene other than Isl2 or Nr2f1b.

Carboxypeptidase N is a plasma metalloprotease and is composed of 2 catalytic activity subunits (cpn1) and 2 regulatory subunits (cpn2). Carboxypeptidase N is critical for zebrafish liver development^20^. Moreover, it has been reported that carboxypeptidase N is responsible for the C-terminal cleavage of SDF-1a in the circulation^21^. SDF1a and its receptor, CXCR4, are key regulators of the hematopoietic, nervous, and cardiovascular systems during embryogenesis, which is essential for hematopoiesis, lymphocyte homing, pre-B-cell growth, and angiogenesis^26, 27^. However, no study has yet reported the role of Cpn1 in vascular function. Thus, our study is the first to report the contribution of *cpn1* in vascular development and we hypothesize that *cpn1* might contribute vascular growth by controlling SDF1a-cxcr4 signals. In this study, we also revealed that *cpn1* regulates vascular development mediated by distinct signals at different localizations in zebrafish. We found *cpn1* expression is down regulated in the trunk region when VEGF or Notch pathways are inhibited whereas it is ectopically expressed under the same conditions in the tail region. These results are consistent with our data that loss of *cpn1* causes ISV growth defects in the trunk while ectopically expression of *cpn1* in the tail likely interferes BMP signals to inhibit EC sprouting and migration at CVP. However, the substrates/targets of Cpn in those signals remain to be elucidated.

Carboxypeptidase N is also known as kininase-1 and anaphylatoxin inactivator and is crucial in the regulation of peptides such as bradykinins and anaphylatoxins. The peptide bradykinin dilates blood vessels and thus reduces blood pressure. Angiotensin-converting enzyme (ACE) inhibitors, a class of anti-hypertensive drugs, lower blood pressure by stabilizing bradykinin^28–30^. However, the regulation of bradykinin by CPN is not fully described. Thus, the relationship among Cpn1, bradykinin, and hypertension (HTN) should be investigated in the future, and Cpn1 can potentially serve as a biomarker for HTN. Mutations in *cpn1* have been associated with angioedema or chronic urticaria caused due to carboxypeptidase N deficiency^31, 32^. Rapidly progressing angioedema is treated as a medical emergency because of the risk of suffocation. However, mechanistic reasons and their regulatory pathways remain unknown. One possible mechanism is that CPN1 deficiency causes the dilation of blood vessels because of the high level of bradykinin accumulation. Long-term dilation of blood vessels can cause angioedema. Meanwhile, dilated vessels can increase immunocyte penetration, and a complement system can result in urticaria. HTN is a major risk factor for cardiovascular diseases and is probably caused by complex interactions among genetic and environmental factors. Angioedema is the rapid allergic swelling of the dermis and subcutaneous tissues. Our study results facilitated an understanding of molecular mechanisms underlying the association of *cpn1* with HTN and angioedema. Thus, we hypothesized that prolonged *cpn1* deficiency and *cpn1* overexpression in animals can respectively cause HTN and angioedema and this hypothesis will be tested in future studies. The use of zebrafish as a model allows for the generation of transgenic or knockout animal models for studying *cpn1*-related HTN and angioedema. Thus, *cpn1* might be used as a novel biomarker and therapeutic target for HTN and angioedema.

Our study highlights new pathways and molecular mechanisms leading to vascular patterning by carboxypeptidase N. Studies on vascular development have high potential medical relevance. Cardiovascular diseases are a major cause of death worldwide. Embryonic vascular development has applications in human congenital and acquired vascular diseases. and some zebrafish vascular mutants have been characterized as models for human congenital disorders. Our results show that, in clinical practice, *cpn1* can be a potential biomarker or therapeutic target for vascular diseases such as HTN, angioedema, and urticaria.

## Methods

### Zebrafish husbandry

Zebrafish (*Danio rerio*) wildtype Tupfel Long Fin (TL) or the transgenic (*Tg*) lines *Tg* (*kdrl*:*eGFP*)^*la116*^, *Tg* (*kdrl*:*mCherry*)^*ci5*^, *Tg* (*gata*:*dsRed*)^*sd2*^, *Tg* (*fli1a*:*egfp*)^*y1*^, and *Tg* (*fli1a*:*negfp*)^*y7*^ have been described^33–37^. Fish and embryos were raised and maintained at 28.5 °C under a 14-h light/10-h dark photoperiod according to the zebrafish book guildline^38^ with approval from the National Sun Yat-sen University Animal Care Committee (approval reference #10231).

### Chemical treatment

Endogenous pigmentation was blocked by adding 0.003% N-phenylthiourea (PTU; Sigma) to E3 media at 6hpf. The embryos were treated with the VEGF inhibitor SU5416 (15 μM, Calbiochem), Notch signal inhibitor DAPT (75 μM, Sigma), and protamine (0.01 mg/mL, Sigma) at a working concentration in E3 medium at 6 hpf. To block BMP signals, the embryos were treated with dorsomorphin (DM, 10 μM, Calbiochem) and DMH1 (40 μM, Calbiochem) at a working concentration at 20 hpf. The control embryos were treated with an equivalent concentration of 0.3% DMSO.

### *In situ* hybridization

Whole-mount *in situ* hybridization was performed according to the method described in refs 39, 40. Probes for *cpn1* were obtained through PCR by using primers listed in Table S1 and through *in vitro* transcription by using T7 Polymerase (Roche) with digoxigenin-labelled UTP. The probes *flk*, *flt4*, *mrc1*, *stabilin*, and *ephrinb2* have been described^18, 33^. Briefly, the embryos were fixed in 4% paraformaldehyde and stored at −20 °C in methanol until use. After rehydration, permeabilization, probing with *target* mRNA overnight, washing, and blocking with 1% BSA, an AP-conjugated antidigoxigenin antibody was added and subsequently reacted with the NBT/BCIP substrate (Roche). The stained embryos were embedded in 3% methylcellulose and photographed using the Zeiss Axiocam HR camera (Carl Zeiss) on the Zeiss Lumar V12 stereomicroscope. For transverse sections, the embryos were fixed in the tissue freezing medium Tek OCT Compound and sectioned at 10 µm by using the Leica CM3050S cryostat and photographed on the IX71 inverted microscope (Olympus) by using the SPOT RT3 camera (Diagnostic Inc.).

### Confocal imaging

For confocal images, the embryos were immobilized and embedded in 1.5% low-melting-point agarose with 5% tricaine (Invitrogen), and images were obtained on the Zeiss LSM700 or Nikon Eclipse 90i C1 confocal microscopes and processed using the ImageJ software (NIH, USA). The counting area was between the 5^th^ and 15^th^ ISVs of the 24–72-hpf embryos. The number of cells in the ISVs was determined by counting cells in the individual slices of confocal stacks. Final figures were processed using Adobe Photoshop.

### Morpholino and Tol2 DNA Injections

Morpholinos for each gene were obtained from Gene-Tools, LLC (Philomath, OR). The following sequences of morpholino oligonucleotides were used:

*p53* MO: 5′-GCGCCATTGCTTTGCAAGAATTG-3′

Standard control MO sequence: 5′-CCTCTTACCTCAGTTACAATTTATA-3′

*cpn1* ^ATG^ MO (5ʹ-GAGCTGCCTGACAGCATGGTCCCA-3ʹ) to block translation and

*cpn1* ^i1e1^ MO (5ʹ-AGAGTAAACACAAGACTCACATGTT-3ʹ) to target the boundary of the intron 1 and exon 1 to interfere with its splicing. The microinjections of morpholino oligonucleotides were administered as previously described. Briefly, morpholinos or expression vectors were injected into the embryos in the 1–2-cell stage on a 3% agar plate. After injections, the embryos were cultured in E3 buffer until examination.

To generate mRNA- or *fli1*:*cpn1*-overexpressing embryos, we used Tol2kit vectors with the multisite Gateway cloning system (Invitrogen) to build expression constructs. The *cpn1*-coding region flanking with *attb1/b2* sequences was amplified from cDNA by using primers listed in Table S1 to generate pDONR-*cpn1*. A Gateway-compatible 0.8-kb *fli1* promoter has been described^41^. Approximately 100 pg of plasmid DNA was coinjected with 40 pg of transposase mRNA into embryos at the 1-cell stage. The success of transgenesis was verified in transient (*fli1:cpn1*)-overexpressing embryos that express cardiomyocyte light chain 2 (*cmlc2*) driven GFP from the pDEST-Tol2-CG2 vector backbone. In addition, capped and polyadenylated mRNA of *cpn1* was synthesized using *in vitro* mMESSAGE mMASCHINE transcription kit with SP6 RNA polymerase (Ambion).

### RNA extraction and quantitative real-time PCR

Total RNA was extracted from approximately 50 embryos at designated stages by using the RNeasy mini kit (Qiagen), and cDNA was prepared using Reverse Transcriptase and oligo-dT primer (Roche) according to manufacturer instructions. Quantitative real-time PCR was performed using the LightCycle 96 instrument (Roche) with SYBR Green I Master (Roche). The qRT-PCR primers for zebrafish are listed in Table S1. Relative gene expression levels were analyzed using the ΔΔ C_t_ method, and *β-actin* was used as a reference gene. All experiments were performed in biological triplicates from three independent batches.

### Acridine orange staining and TUNEL assay

The dechorionated embryos were soaked in E3 medium containing 2 µg/ml acridine orange for 30 min. After washing 6 times with fresh E3 medium, the embryos were mounted in 3% methylcellulose with 5% tricaine and photographed.

TUNEL assay was performed to analyze apoptosis in the embryos. The 30 hpf embryos were fixed in 4% paraformaldehyde overnight, dehydrated in methanol, and stored at −20 °C. The embyros were rehydrated and treated with 10 µg/ml proteinase K for 20 min at room temperature before labelling DNA breaks with terminal deoxynucleotidyl transferase and fluorescein-dUTP (Roche) according to manufacturer instructions. For fluorescence signal detection, the embryos were blocked in 5% sheep serum (SS), incubated with peroxidase-conjugated antifluorescein antibody (diluted 1:2000; Roche) overnight at 4 °C, washed, and visualized using the DAB color development kit (Roche).

### Protein Extraction and Western Blotting

Embryos lysates were prepared using RIPA lysis buffer (50 mM Tris-HCl pH 7.4, 1% NP-40, 0.25% sodium deoxycholate, 150 mM NaCl, 1 mM PMSF and protease inhibitors) and phosSTOP (Roche). The lysates were spin down, added SDS sample buffer and boiled for 10 mins. The proteins were separated by SDS-PAGE and then transferred to a polyvinylidene fluoride (PVDF) membrane (Bio-rad). The membranes were washed in TBS (Tris-buffered saline) with Tween-20 (TBST) and blocked with nonfat milk in TBST for 1 hr. The membranes were then incubated with anti- phospho histone H3 (pHH3) (Millipore), anti-Histone H3, anti-Erk1/2 and anti-phospho-Erk1/2 (Cell Signaling Technology), anti-PCNA, anti-Samd1/5/8 and anti-phospho-Samd1/5/8 (Santa Cruz Biotechnology) polyclonal antibodies and anti-β-actin monoclonal antibody (Sigma) overnight at 4 °C. The membranes were washed with TBST and incubated with a peroxidase conjugated secondary antibody (Cell Signaling Technology) at room temperature for 2 h. The signal was detected by ECL chemiluminescence (Bio-Rad).

### Anti-phospho histone H3 staining

Embryos were fixed with PFA, permeabilized with pre-chilled acetone for 10 min, washed in PBS with 0.1% Tween-20 (PBSTw), and blocked with 10% normal goat serum (NGS) in PBS with 0.8% Triton X-100 (PBSTx) for 1 h at room temperature. Then, embryos were incubated overnight at 4 °C in the anti-phospho histone H3 polyclonal antibody (Millipore; 1:300 in 1% NGS/PBSTx). After washing 4 times for 10 min each in PBSTw, the embryos were incubated for 2 h at room temperature in the dark with the secondary antibodies (donkey anti-rabbit IgG-CFL 555, Santa Cruz #sc-362271; 1:200 in 1% NGS-PBSTx). Embryos were washed 5 times, embedded in 3% methylcellulose, and photographed using the Zeiss Axiocam HR camera (Carl Zeiss) on the Zeiss Lumar V12 fluorescent stereomicroscope.

## Electronic supplementary material

Supplementary information

Dataset 1
