# Supplementary material for: Fine-tune regulation of carboxypeptidase N1 controls vascular patterning during zebrafish development
Source: Sci Rep. 2017 May 12;7:1852. doi: 10.1038/s41598-017-01976-x (PMC5431830; doi:10.1038/s41598-017-01976-x)
Supplement: Supplementary file 1 — Supplementary information [file 41598_2017_1976_MOESM1_ESM.pdf]

Supplementary Information

Fine-tune regulation of carboxypeptidase N1 controls vascular patterning during zebrafish development

Ting-Yun Wu[1,+], Yi-Shan Wang[1,+], Yi-Chun Song[1], Zih-Ying Chen[1], Yi-Ting Chen[1], Chien-Chih Chiu[1,4], and Chang-Yi Wu[1,2,3,4]*

Methods

Morpholino efficiency

The efficiency of morpholinos, which cause mis-splicing or reduce band signals, was determined through PCR by using primers flanking on the exon 1 and exon 2. RNAs extracted from splicing morpholinos injected and uninjected embryos and reverse transcript to cDNA as templates. The primers cpn1 MO_f :5′-AGGAGATGGTGAGGGCTCTT-3′ and cpn1 MO_r: 5′-TCAGGGTTCATGGAAGGAAG-3′ were used to examine the efficiency of *cpn1* knockdown, and the primers GAPDH_f :5′-TGCTGTAACCGAACTCATTGTC-3′ and GAPDH_r :5′-CAAGCTTACTGGTATGGCCTTC-3′ were used as a loading control.

Cpn1-atg-GFP fusion construct and mRNA synthesis

Polymerase chain reaction (PCR) was used to amplify 150bp fragment around *cpn1* translational start site contained *cpn1* atg morpholino binding region. The primers are 5’-AGTCGGATCCAGGAAAAGAAGTGGCGCTCT-3’ and 5’-AAGTCCATGGGAAGCCTCGAGCCCCAGAAG-3’. The resulting *Bam*HI/*Nco*I digested fragments were subcloned into pCS2-GFP to generate the plasmid pCS2-cpn1-atg-GFP fusion construct. The *cpn1-atg-GFP* fusion construct was linearized with *Not*I and SP6 RNA polymerase was used for in vitro synthesis of capped mRNA (Ambion).

Table S1: Primer sequences used in this study

Gene	Primer sequences
cpn1_f	5′-TCAGAAGCTGGCAAAGACA-3′
cpn1_rT7	5′-TAATACGACTCATATAGTGAAAAACCCGCTTTTATGC -3′
cpn1-f1-attb1	5′-GGGGACAAGTTTGTACAAAAAAAGCAGGCTCCACC ATGCTGTCAGGCAGCTCTCTC-3′
cpn1-r1-attb2	5′-GGGGACCACTTTGTACAAGAAAGCTGGGTTTAATTCTT GGGCCAAGGTTGGTG-3′
cpn1	cpn1-qf:5′-GGAAGGGAGCTGCTCATCTA-3′<br>cpn1-qr:5′-TCAGGGTTCATGGAAGGAAG-3′
dab2	dab2-qf:5′-CATTCCAGCAGACCTGTTCA-3′<br>dab2-qr:5′-CCCAAGAGACTGGAGCTGTC-3′
ef1a	ef1a-qf:5′-CCTTCGTCCCAATTTCAGG-3′<br>ef1a-qr:5′-CCTTGAACCAGCCCATGT-3′
ephrinb2	ephrinb2-qf:5′-CTGGAACACCACGAACACC-3′<br>ephrinb2-qr:5′-CACACGTGGGCAAACTATGT-3′
fli1a	fli1a-qf:5′-CCGAGGTCCTGCTCTCACAT-3′<br>fli1a-qr:5′-GGGACTGGTCAGCGTGAGAT-3′
flt4	flt4-qf:5′-ACTCGGGTTATTACCGCTGCTTCT-3′<br>flt4-qr:5′-TGGATGCTCTGGGTCTCGAACAAA-3′
gridlock	gridlock-qf:5′-GGCTCACCTACAACGACATCC-3′<br>gridlock-qr:5′-CCAACTTGGCAGATCCCTGT-3′
flk	flk-qf:5′-ACTTTGAGTGGGAGTTTCATAAGGA-3′<br>flk-qr:5′-TTGGACCGGTGTGGTGCTA-3′
mrc1	mrc1-qf:5′-CTAGCAAGCCTGAAGGTGCC-3′<br>mrc1-qr:5′-TGAGAGGCTGGGTAGTTGGG-3′
stabilinb2	stabilinb2-qf:5′-GGGCTTCCAATACCAACTGG-3′<br>stabilinb2-qr:5′-CCTGGTTGCACAGACAGACC-3′
β-actin	β-actin_qf:5′-CTCTTCCAGCCTTCCTTCCT-3′<br>β-actin_qr:5′-CTTCTGCATACGGTCAGCAA-3′

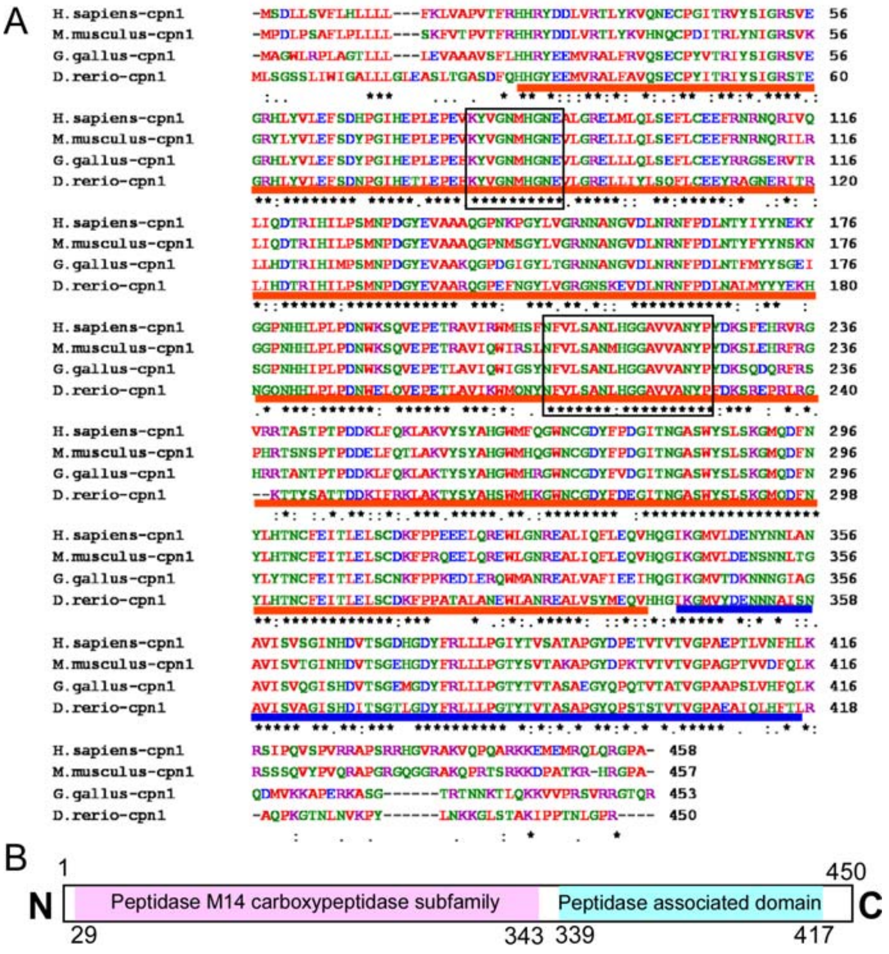

C

H.sapiens-cpn1	M.musculus-cpn1	G.gallus-cpn1	D.rerio-cpn1	
100.00	83.59	70.58	67.26	H.sapiens-cpn1
	100.00	69.84	65.84	M.musculus-cpn1
		100	69.28	G.gallus-cpn1
			100	D.rerio-cpn1

Figure S1. Sequence alignment zebrafish Cpn1 with other species.
(A) Alignment of amino acid sequences of the human (*H. sapiens*, GenBank accession number NP_001299.1), mouse (GenBank accession number NP_109628.1), chicken (GenBank accession number NP_001264183.1), and zebrafish Cpn1 protein (GenBank accession number NP_997935.1) by using the ClustalW2 software. Identical amino acids are marked (*) below the sequence. The M14 family of metallocarboxypeptidases (orange) and peptidase-associated domain (blue) are boxed. (B) The schematic drawing shows that zebrafish Cpn1 protein contains 450 amino acids with the N-terminal M14 family of metallocarboxypeptidase domain and C-terminal peptidase-associated domain. (C) Comparison of the percentage of Cpn1 amino acid identity among human, mouse, and zebrafish.

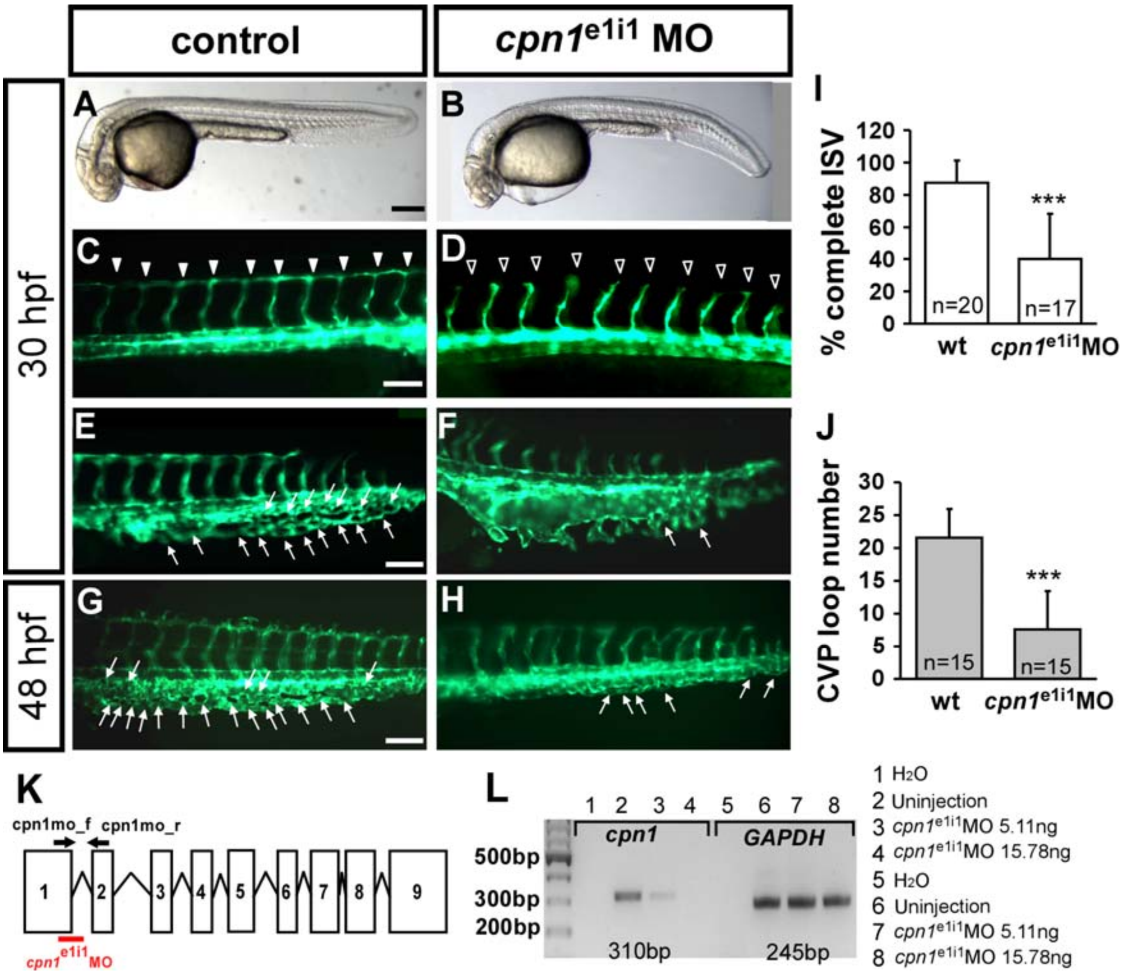

Figure S2. Specificity of *cpn1* knockdown by splicing morpholino.
(A–H) The knockdown of *cpn1* by splicing MO caused similar defects in the vasculature, including defects in ISV growth *(hallow arrowheads* in **D***)* and less CVP sprouting **(F)** and loop formation **(H)** *(arrows)* compared to wild-type control. **(I)** The percentage of completed ISVs decreased by about 47% in *cpn1*^{e1i1} morphants (n = 20 in wt and n = 17 in *cpn1*^{e1i1} MO) at 30 hpf. **(J)** The loop formation in CVP exhibited a decrease number 7.6 ± 5.8 in *cpn1*^{e1i1} morphants compared to wt control (21.6 ± 4.4) (n = 15 in wt and *cpn1*^{e1i1} MO) at 48 hpf. **(K)** The scheme presents *cpn1*^{e1i1} morpholino targeting the pre-mRNA structure of *cpn1*, suggesting the missplicing of a fragment, which can be detected using a cpn1mo_f and cpn1mo_r primer set. **(L)** Total RNA from controls or *cpn1* morphants (injected with 5.11 ng or 15.78 ng *cpn1*^{e1i1} morpholino) was used in RT-PCR with primers for the *cpn1* gene. In morphants injected with *cpn1*^{e1i1} morpholino, GAPDH levels remain unchanged, whereas the amount of *cpn1* product (310 bp) decreased with an increase in the *cpn1*^{e1i1} morpholino dose. The data suggest that *cpn1*^{e1i1} morpholino-knockdown specifically targets *cpn1*.

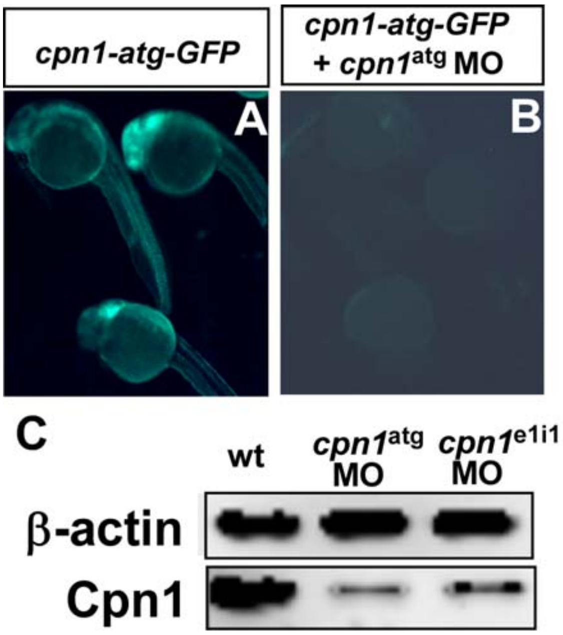

Figure S3. Targeting specificity of *cpn1* ATG morpholinos.
(A) At 24hpf, embryos injected with mRNA from the *cpn1-atg-GFP* construct results in GFP expression (*n*=24/25 with GFP). (B) Co-injection of (*cpn1-atg-GFP***)** mRNA and *cpn1* MO reduced and/or blocked the green signal in the embryos (*n*=2/22 with GFP). The images are the representative pictures from two independent experiments. (C) Western blot analysis showed the reduction of Cpn1 protein levels significantly by morpholino knockdown of *cpn1* to block translation or to interfere with *cpn1* splicing. Rabbit polyclonal anti-Cpn1 antibody (TA340008) is purchased from OriGene and β-actin serves as a loading control. The data shown is representative of two independent experiments.

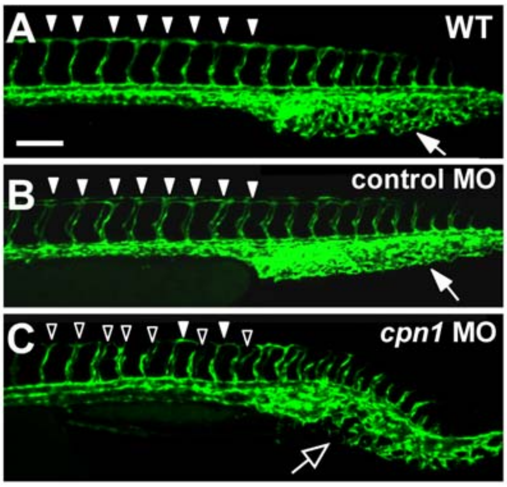

Figure S4 Injection of control morpholino does not cause vascular defects.
(A-C) Confocal images analysis shows vascular development in ISV and CVP at 30 hpf in wild type uninjected control **(A)**, standard negative control morpholino injected embryos **(B)** and injected with *cpn1* translation blocking morpholinos **(C).** ISV and CVP was unaffected by control morpholino injection (*arrow* and *arrowheads*). *Cpn1* morphants show a short or incompletion of ISVs (*hollow arrowheads*) and lack of CVP loops (*arrow*). The scale bar for **A-C** represent 100 μm.

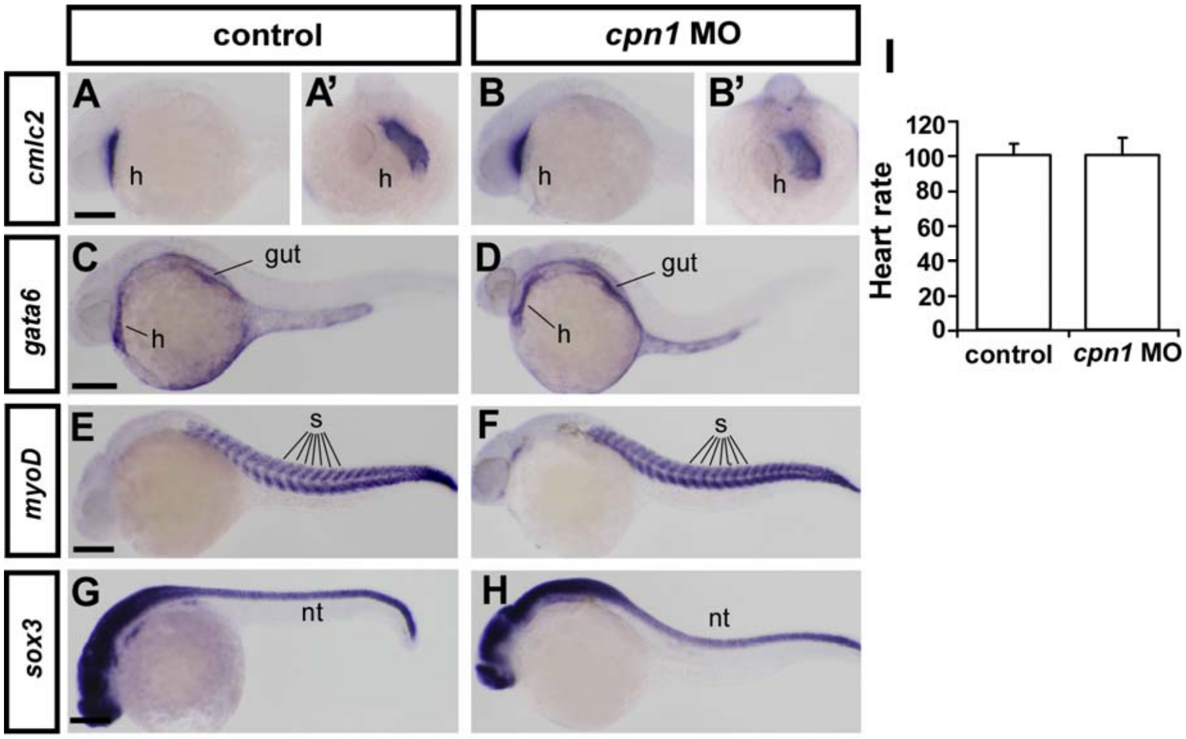

Figure S5. Gross developmental process is unaffected in *cpn1* morphants.
Knockdown of *cpn1* did not alter the expression pattern of heart, gut, somite and neural systems by examining the expression of *cmlc2* (heart marker, h; **A-B** are lateral view and **A'-B'** are ventral view), *gata6* (heart and gut marker, **C, D**), *myoD* (somite marker, s, **E, F**), and *sox3* (neural tube marker, nt, **G, H**). Those probes have been described and documented in ZFIN. **(I)** Knockdown of *cpn1* did not cause developmental delay by measuring heart rate. Quantification of zebrafish heart beats per minute in uninjected control and *cpn1* morphants shows no difference (n=16 in control and n=19 in *cpn1* MO) at 24-25hpf. The mean heart rate determined by direct visual examination of ventricle beating in control was 100.7 ± 6.2 beats per minute, 100.4 ± 10.3 beats per minute in *cpn1* MO. The scale bars represent 250 μm in **A-H.**

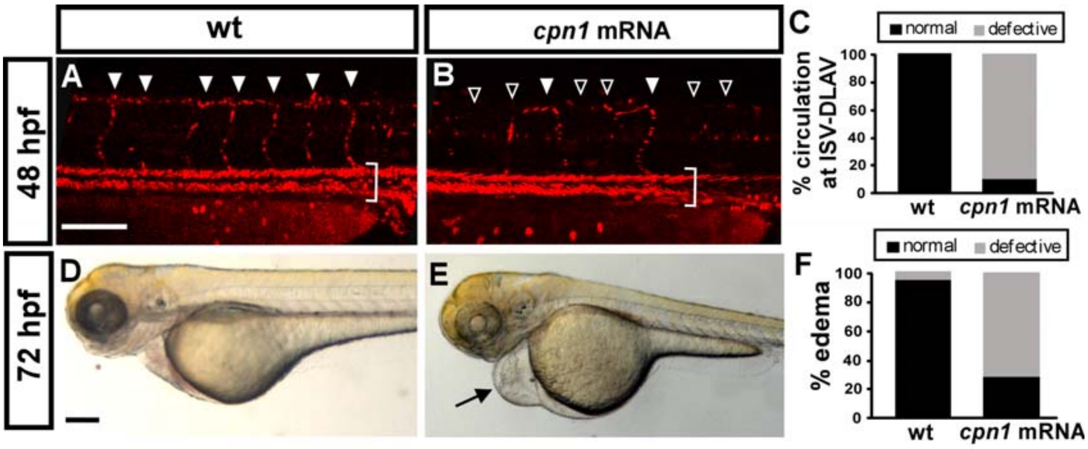

Figure S6. Overexpression of *cpn1* causes circulation defects and pericardial edema.
(A-C) Injection of *cpn1* mRNA into *Tg(fli:eGFP^{y1} ; gata1:dsRed)* embryos causes circulation defect at ISV-DLAV at 48hpf. Quantification data showed overexpressing cpn1 embryos with 90% defects in circulation compared to control (n=10 for both conditions). **(D-F)** At 72hpf, overexpresion of *cpn1* shows pericardial edema (*arrow* in **E**). Quantification of percentage of pericardial edema showed ~70 % increase in *cpn1* mRNA injected embryos compared to control (n=33 in control and n=28 in *cpn1* mRNA injection) at 72hpf. Scale bars are 100 µm for A, B and 200 µm for D, E.

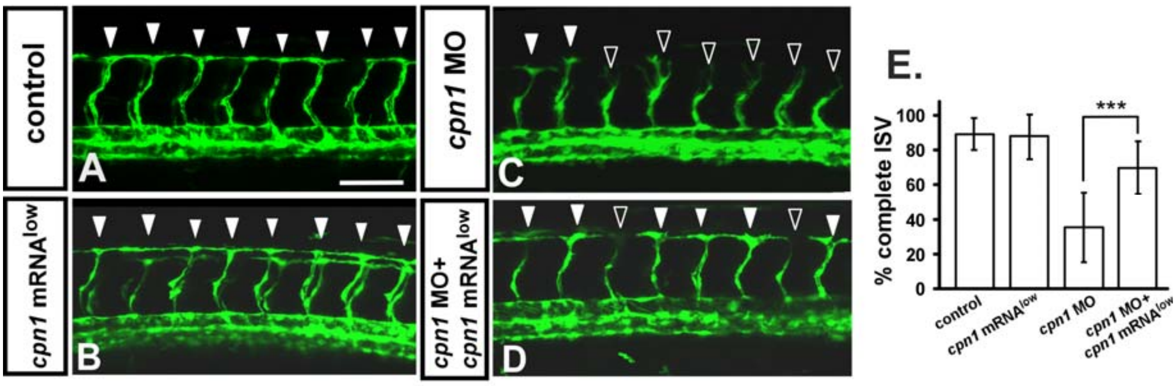

Figure S7. Knockdown of *cpn1* can be rescued by overexpression of *cpn1*.
In uninjected control embryos, ISV has reached the dorsal region and formed DLAV around 28–30 hpf ((**A**), *arrowheads*). At the same stage in *cpn1* MO, ISVs have stalled or slowed growth at mid-somite ((**C**), *hollow arrowheads*). Overexpression of *cpn1* by *cpn1*mRNA (100pg) injection had no obvious defect in vasculature (**B**), but rescued the defect of ISV stalling ((**D**), *solid arrowheads*). (**E**) Quantification of percentage of completed ISV at 30 hpf shows a ~35% increase in rescued embryos compare to *cpn1* morphants. Percentages of completed ISV are ~90 ± 9, 88 ± 13, 35 ± 20, and 70 ± 15 in control, *cpn1* mRNA overexpression, *cpn1* MO, and rescued embryos, respectively. (*** refers to $p < 0.0001$ by an unpaired Student's *t*-test. Data represent means ± S.D). Scale bars are 100 µm for (**A–D**).

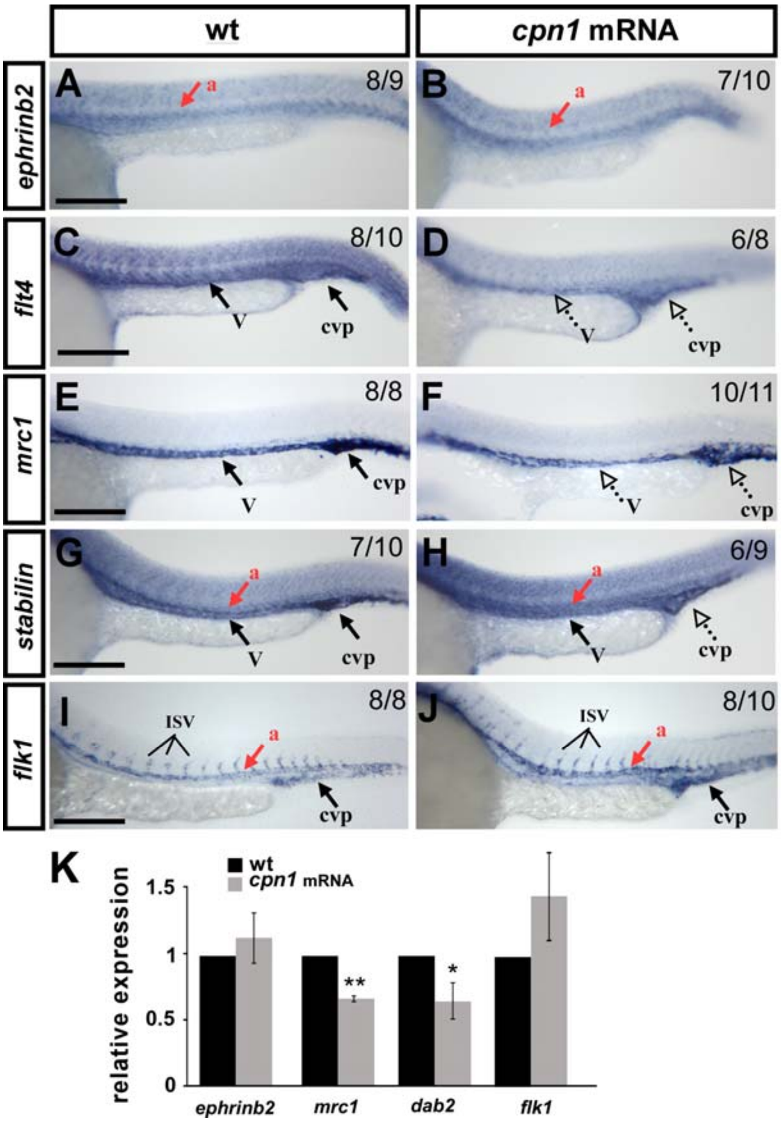

Figure S8. Overexpression of *cpn1* alters the expression of vascular markers.
The expression of vascular markers was examined by in-situ hybridization **(A-J)** and/or quantitative real-time PCR **(K).** Compared to the wild type controls at 24 hpf **(A, C, E, G, I),** the expression of the venous/ISV markers *flt4* **(D, K)**, *mrc1* **(F, K)** and *dab2* **(K)** was decreased in overexpressed *cpn1* embryos (*cpn1*mRNA); the expression of the pan-vascular markers *stabilin* **(H),** and *flk* **(J, K)** was no-to-slightly increased in embryo; the expression of the arterial marker *ephrinb2* is unchanged **(B, K)**. (K) Quantitative results by qPCR showed the expression of vascular markers *ephrinb2* (1.1 ± 0.2), *mrc1* (0.64 ± 0.05), *dab2* (0.62 ± 0.12) and *flk1* (1.4 ± 0.3),) in overexpressing *cpn1* embryos. Values on the top right indicate the number of embryos exhibiting phenotype per total number of embryos analyzed. Scale bars are 200 µm. dorsal aorta (a); vein (v), and caudal vein plexus (cvp). Data are represented as means ± S.D. ** refers to $p < 0.001$ and * refers to $p < 0.05$ by an unpaired Student's *t*-test.
