# Supplementary material for: Fine-tune regulation of carboxypeptidase N1 controls vascular patterning during zebrafish development
Source: Sci Rep. 2017 May 12;7:1852. doi: 10.1038/s41598-017-01976-x (PMC5431830; doi:10.1038/s41598-017-01976-x)
Supplement: Supplementary file 2 — Dataset 1 [file 41598_2017_1976_MOESM2_ESM.doc]

**Supplementary dataset**

**Fine-tune regulation of carboxypeptidase N1 controls vascular patterning during zebrafish development**

Ting-Yun Wu^1,+^, Yi-Shan Wang^1,+^, Yi-Chun Song^1^, Zih-Ying Chen^1^, Yi-Ting Chen^1^,

Chien-Chih Chiu^1,4^, and Chang-Yi Wu^1,2,3,4^*

This supplementary dataset contains the original full-length Western blots, which the display of cropped blots in Figure 3Q, Figure 7T and sFig. 3C.


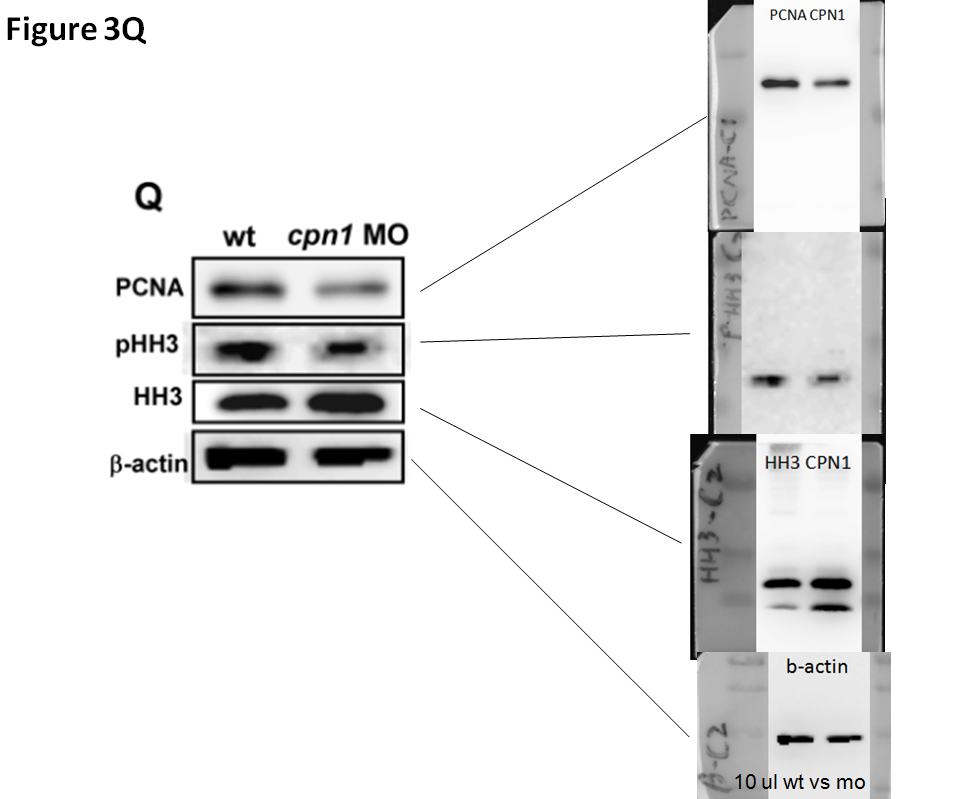


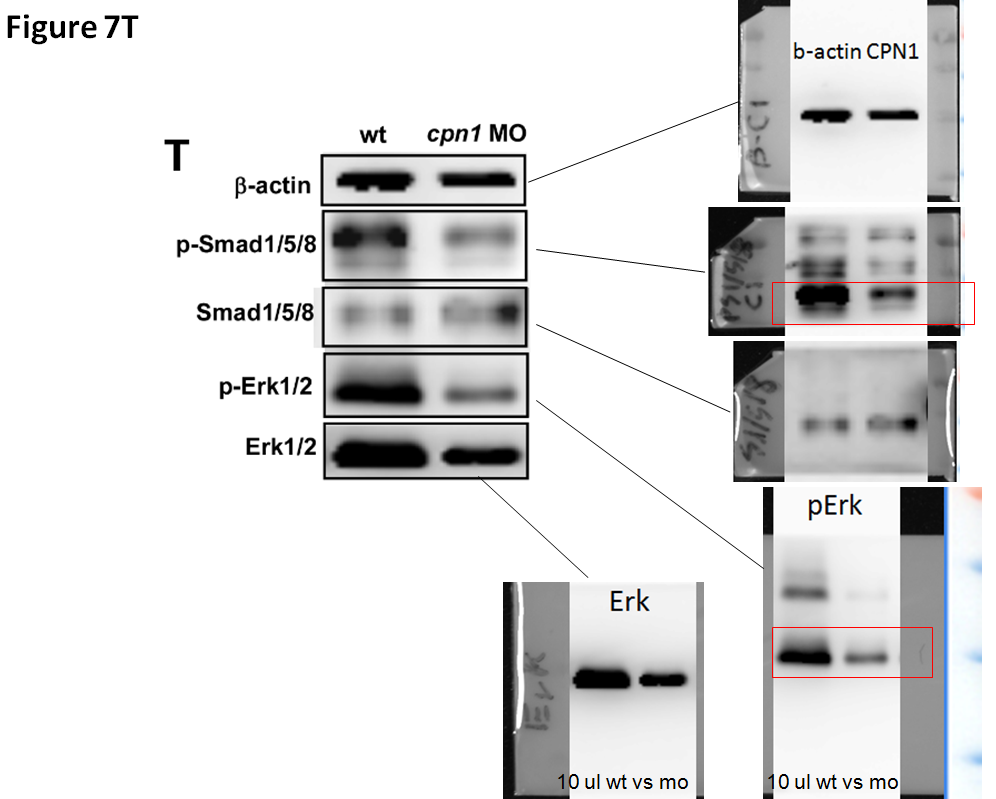


**
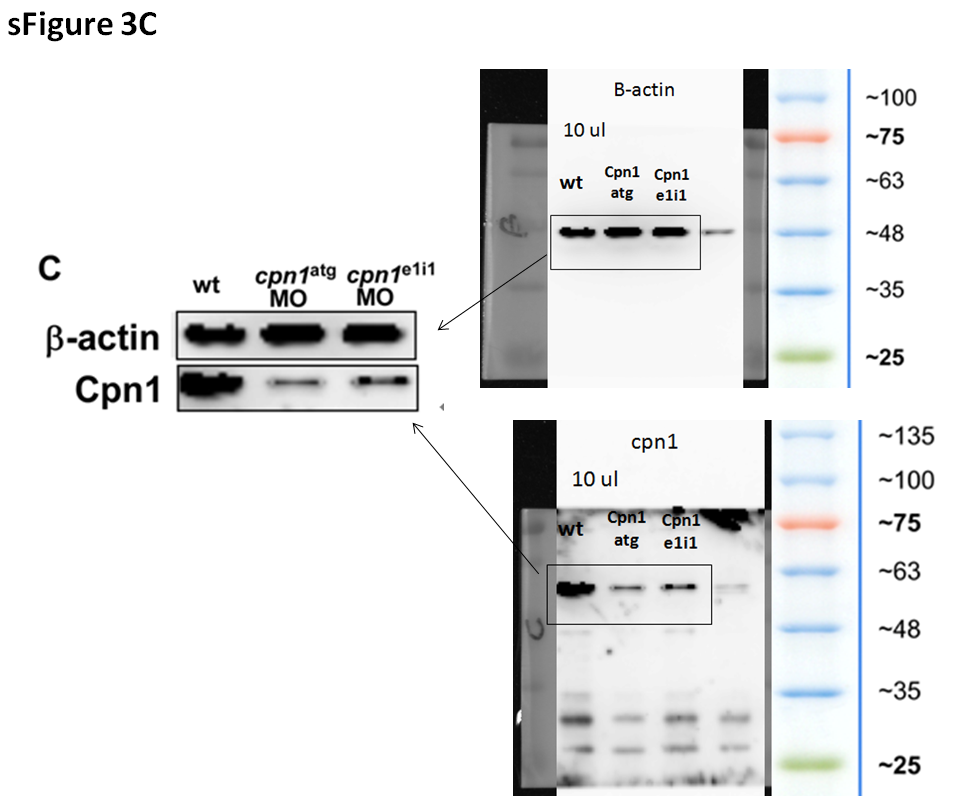
**
